# Combining population genomics and forward simulations to investigate stocking impacts: A case study of Muskellunge (*Esox masquinongy*) from the St. Lawrence River basin

**DOI:** 10.1111/eva.12765

**Published:** 2019-01-30

**Authors:** Quentin Rougemont, Anne Carrier, Jeremy Le Luyer, Anne‐Laure Ferchaud, John M. Farrell, Daniel Hatin, Philippe Brodeur, Louis Bernatchez

**Affiliations:** ^1^ Département de biologie, Institut de Biologie Intégrative et des Systèmes (IBIS) Université Laval Québec Québec Canada; ^2^ IFREMER, Unité Ressources Marines en Polynésie, Centre Océanologique du Pacifique Taravao, Tahiti French Polynesia; ^3^ Department of Environmental and Forest Biology, College of Environmental Science and Forestry State University of New York Syracuse New York; ^4^ Ministère des Forêts, de la Faune et des Parcs, Direction de la Gestion de la Faune Estrie‐Montréal‐Montérégie‐Laval Longueuil Québec Canada; ^5^ Ministère des Forêts, de la Faune et des Parcs Direction de la gestion de la faune de la Mauricie et du Centre‐du‐Québec Trois‐Rivières Quebec Canada

**Keywords:** admixture, *Esox masquinongy*, forward simulations, gene flow, genomic, stocking

## Abstract

Understanding the genetic and evolutionary impacts of stocking on wild fish populations has long been of interest as negative consequences such as reduced fitness and loss of genetic diversity are commonly reported outcomes. In an attempt to sustain a fishery, managers implemented nearly five decades of extensive stocking of over a million Muskellunge (*Esox masquinongy*), a native species in the Lower St. Lawrence River (Québec, Canada). We investigated the effect of this stocking on population genetic structure and allelic diversity in the St. Lawrence River in addition to tributaries and several stocked inland lakes. Using genotype by sequencing, we genotyped 643 individuals representing 22 locations and combined this information with forward simulations to investigate the genetic consequences of long‐term stocking. Individuals native to the St. Lawrence watershed were genetically differentiated from stocking sources and tributaries, and inland lakes were naturally differentiated from the main river. Empirical data and simulations within the St. Lawrence River revealed weak stocking effects on admixture patterns. Our data suggest that the genetic structure associated with stocked fish was diluted into its relatively large effective population size. This interpretation is also consistent with a hypothesis that selection against introgression was in operation and relatively efficient within the large St. Lawrence River system. In contrast, smaller populations from adjacent tributaries and lakes displayed greater stocking‐related admixture that resulted in comparatively higher heterozygosity than the St. Lawrence. Finally, individuals from inland lakes that were established by stocking maintained a close affinity with their source populations. This study illustrated a benefit of combining extensive genomic data with forward simulations for improved inference regarding population‐level genetic effects of long‐term stocking, and its relevance for fishery management decision making.

## 1 INTRODUCTION

Many native species are undergoing steep population declines due to rapid and globally changing environments and overexploitation (Allendorf, 2017). In response, supplementation programs continue to be extensively applied to enhance natural populations to sustain exploitation and more recently for biodiversity conservation (Laikre, Schwartz, Waples, Ryman, & GeM Working Group, 2010). Fish populations, in particular, are subjected to intense commercial and recreational exploitation (Dunham, 2011). This leads to species transfers, sometimes over large distances, to supplement genetically and ecologically divergent populations. As a consequence, understanding how supplementation practices from nonindigenous sources may impact the evolutionary potential of wild populations remains a major concern (Araki, Cooper, & Blouin, 2007, 2009; Laikre & Ryman, 1996; Ryman & Laikre, 1991; Waples, Hindar, Karlsson, & Hard, 2016).

From a genetic standpoint, the potential negative impacts of stocking include the following: reduction in genetic diversity and effective population size due to the Ryman–Laikre effect (i.e., use of small numbers of individuals for breeding programs, Laikre & Ryman, 1996; Ryman & Laikre, 1991), genetic homogenization of wild populations (Araki & Schmid, 2010; Eldridge, Myers, & Naish, 2009; Eldridge & Naish, 2007; Lamaze, Sauvage, Marie, Garant, & Bernatchez, 2012; Perrier, Guyomard, Bagliniere, Nikolic, & Evanno, 2013), and ultimately the loss of locally adapted traits (Araki, Cooper, & Blouin, 2009; Ford, 2002; Lynch & O'Hely, 2001). Loss of local adaptation may arise from selection of traits associated with captive environments or because a divergent stocking source may exhibit reduced fitness in the local environment (Fraser et al., 2018). With admixture (impacting genome‐wide structure) and eventual introgression (where allelic variants are transferred from one differentiated population to another), differences in selective values can lead to outbreeding depression through disruption of co‐adapted gene complexes or from the breakup of epistatic interactions (Lynch, 1991; Tallmon, Luikart, & Waples, 2004). Such results have been documented in several occasions (e.g., Allendorf, Leary, Spruell, & Wenburg, 2001; Edmands, 2007; Le Cam, Perrier, Besnard, Bernatchez, & Evanno, 2015). These have raised concern regarding the consequences of stocking on long‐term maintenance and recovery of wild populations (Létourneau et al., 2018).

Conversely, positive effects of supplementation have also been reported. Several threatened populations recently isolated due to habitat fragmentation often display low genetic divergence and have not accumulated genetic incompatibilities. These small populations, however, are exposed to genetic drift whereby mildly deleterious mutations are not efficiently removed by selection, as would be the case in larger populations (Whitlock & Bürger, 2004). These mildly deleterious mutations may increase in frequency and rise to fixation (Glémin, 2003; Lynch et al., 1999; Wang, Hill, Charlesworth, & Charlesworth, 1999). This random drift load, however, becomes important when the selection coefficient is >50% of the effective population size (Whitlock, 2000) and can ultimately lead to extinctions of very small populations (Lynch, Conery, & Bürger, 1995). Similarly, inbreeding depression is of concern in these small populations (Bataillon & Kirkpatrick, 2000; Wang et al., 1999). In the long term, this may pose major threat to the adaptability of these populations to changing environment (Carlson, Cunningham, & Westley, 2014; Ralls et al., 2018).

Population supplementation, when done appropriately (see Miller et al., 2017), can have beneficial effects (Frankham, 2015; Frankham et al., 2011; Ralls et al., 2018). For instance, Frankham (2015) showed a genetic rescue effect after outcrossing that provided a 148% median fitness increase in wild populations exposed to stressful environments. Such effects have been shown to persist through several generations (Frankham, 2015; Frankham, 2016; Ralls et al., 2018), and, when applied sensibly, the probability of outbreeding depression is generally low (Frankham et al., 2011).

While admixture and introgression have been extensively documented in salmonid species (Finnegan & Stevens, 2008; Hansen, Fraser, Meier, & Mensberg, 2009; Létourneau et al., 2018; Perrier, Baglinière, & Evanno, 2013; Perrier, Guyomard et al., 2013), less emphasis has been placed on other taxonomic groups. Here, we examine genetic implications of long‐term stocking in Muskellunge *Esox masquinongy*, a representative of the Esocidae, the sister family of all salmonids (Rondeau et al., 2014). Muskellunge are distributed within particular temperate rivers and lakes from midwestern to eastern North America (Crossman, 1978), including the Great Lakes where it often occurs in sympatry with its congener, Northern Pike *Esox lucius*. In the St. Lawrence River, Muskellunge reaches sexual maturity between 5 and 7 years for males and 6 and 8 years for females (Farrell et al., 2007) and can grow up to more than 1.5 m and 20 kg that makes it a highly prized species for recreational trophy fishing. Given its typically low population density (~<1.0 fish/ha; Cloutier, 1987; Simonson, 2010) that limits sampling programs, more information is needed regarding its biology, genetic diversity, and population dynamics (Crane et al., 2015; Kapuscinski, Sloss, & Farrell, 2013). In many places, native Muskellunge have undergone pronounced population declines due to various factors including overharvesting, habitat degradation, and pollution (Brodeur, Hatin, & Bacon, 2013; Crossman, 1978; Farrell et al., 2007; Mongeau & Massé, 1976; Whillans, 1979). The decline has been more recently reinforced by viral hemorrhagic septicemia and round goby introduction (Farrell, Getchell, Kapuscinski, & LaPan, 2017).

In response to Muskellunge management challenges, widespread and sometimes intensive and long‐term stocking is used to rehabilitate native populations and supplement existing ones. Stocking was also performed as species introductions to support new recreational fisheries and as a predator to control invasive fish species (Crane et al., 2015; Jennings et al., 2010; Kapuscinski, Belonger, Fajfer, & Lychwick, 2007; Wingate, 1986). Studies regarding Muskellunge genetic divergence and stocking effects primarily used microsatellite markers and mainly focused on the Laurentian Great Lakes and Upper St. Lawrence River (Kapuscinski et al., 2013; Miller, Mero, & Younk, 2009, 2012; Turnquist et al., 2017; Wilson, Liskauskas, & Wozney, 2016). These studies revealed pronounced genetic differentiation among weakly connected populations at small spatial scales. They also demonstrated a generally reduced genetic diversity putatively associated with recent bottlenecks and/or strong genetic drift in populations of small effective size and restricted dispersal due to spawning site fidelity (Crossman, 1990; Farrell et al., 2007; Jennings, Hatzenbeler, & Kampa, 2011; Margenau, 1994; Miller, Kallemeyn, & Senanan, 2001). The impact of stocking has been documented in several populations where modest levels of admixture and a partial homogenization of population genetic structure have been reported (Scribner et al., 2015). In contrast, no study has documented native Muskellunge population structure further east, in the Lower St. Lawrence River basin of Québec (Canada), downstream of the Great Lakes. Moreover, no population genomic studies using thousands of markers distributed throughout the genome have been performed on this species to date.

Similar to populations throughout its distribution range, Lower St. Lawrence Muskellunge experienced a prolonged decline attributed to habitat loss (Robitaille & Cotton, 1992) and commercial overfishing that occurred until 1936 (Crossman, 1986; Dymond, 1939). Therefore, from 1951 to 1998 the Lower St. Lawrence was supplemented by over 1 million young Muskellunge (Brodeur et al., 2013; De La Fontaine, unpublished; Dumont, 1991; Mongeau, Leclerc, & Brisebois, 1980; Vézina, 1977; Vincent & Legendre, 1974). In particular, during 1951–1965, fish from both southwestern New York State (Chautauqua Lake, Ohio River basin) and Ontario's Kawartha Lakes (represented in our study by Pigeon Lake) were transferred to Lachine hatcheries in Québec to support a massive stocking program in the St. Lawrence River (De La Fontaine, unpublished; Dumont, 1991). Muskellunge from the Ohio basin are thought to rise from a distinct regional lineage (Koppelman & Philipp, 1986), but phylogeographic and systematic relationships of these two stocking sources are incompletely understood (Miller et al., 2017). Fish from those sources were also stocked in numerous lakes including Lake Joseph and Lake Tremblant, which themselves became widely used as a brood source from 1965 for hatcheries until the end of stocking in 1998 (De La Fontaine, unpublished; Dumont, 1991; Vézina, 1977; Vincent & Legendre, 1974). Besides stocking in those waterbodies, Muskellunge were also introduced in several lakes and rivers (details on stocking history and translocations are provided in Supporting Information Figure S1).

An improved understanding of the effects of admixture following stocking can also be advanced by application of individual‐based forward simulations (Hoban, 2014; Hoban, Bertorelle, & Gaggiotti, 2012). Simulated outcomes of population divergence from a variety of demographic scenarios (e.g., in terms of population size and differential mortality) may be used to identify the one that best explains empirically documented patterns (Guillaume & Rougemont, 2006; Hernandez, 2008; Lawrie, 2017; Messer, 2013). These methods, however, are rarely applied to investigate stocking effects on the genetic composition of wild fish (but see Perrier, Guyomard et al., 2013). It is also possible to generate increasingly complex and biologically realistic simulations for comparison with empirical data. Such simulations can aid in understanding supplementation efficacy and how it may generate admixture, while also helping to understand the limits of simple clustering tools (e.g., Alexander, Novembre, & Lange, 2009; Pritchard, Stephens, & Donnelly, 2000) in detection of genome‐wide effects of admixture on local ancestry.

Here, we combined empirical population genomics using a genotype‐by‐sequencing approach with forward simulations to document the extent and patterns of genetic admixture that resulted from past stocking events in the St. Lawrence River watershed. With this dataset and simulation tools, we (a) explored the effect of nearly five decades of stocking on wild populations and documented fine‐scale population genetic diversity and structure of the Muskellunge in the St. Lawrence River watershed, (b) compared expected patterns of admixture from simplified demographic simulations with empirical observations, and (c) used this information for management recommendations pertaining to stocking practices.

## 2 METHODS

### 2.1 Sampling

**Figure 1:**
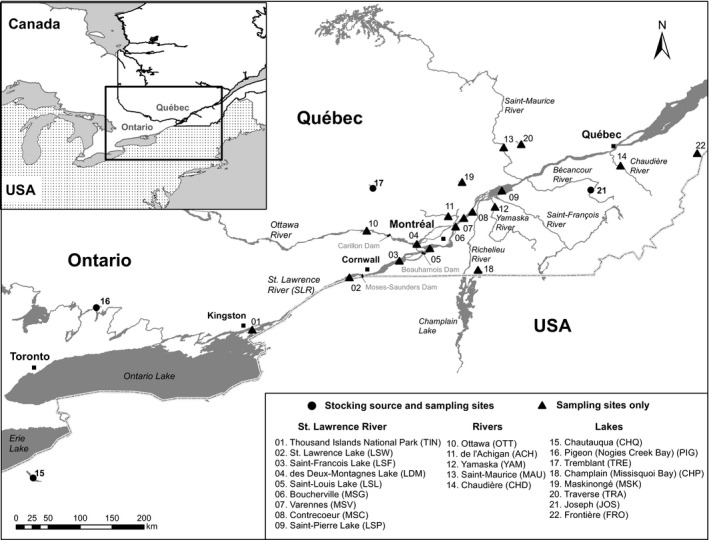
Map showing sampling locations. Two major physical barriers are present on the St. Lawrence system: Moses‐Saunders and Beauharnois‐Les Cèdres hydropower dams located at the upstream and downstream end of Lake Saint‐Françis, respectively

A total of 662 Muskellunge were sampled from 22 sites within the St. Lawrence River drainage, mainly from Québec (Canada) with a median sample size of 24 individuals per site (Figure 1, Table 1). This sampling represents the Upper and Lower St. Lawrence River, its main tributaries, and inland lake populations. Two sampling sites (Chautauqua Lake, CHQ, and Pigeon Lake, PIG) represented the original stocking sources used from 1951 to 1965 and two more recent secondary sources (Lake Joseph, JOS, 1965–1986, and Lake Tremblant, TRE, 1986–1997; both initially stocked with the CHQ and PIG source) were included in the baseline. Of all sampling locations, to the best of our knowledge, only Traverse Lake (TRA) had no record of past stocking and is presumed to be native. Most samples (fin clips preserved in 95% ethanol) were obtained from catch‐and‐release captures of professional fishing guides and anglers between 2010 and 2015. Samples were also collected by the Ministère des Forêts, de la Faune et des Parcs du Québec (MFFP), the Ontario Ministry of Natural Resources and Forestry (MNRF), and the New York Department of Environmental Conservation. A graphical representation of the major stocking operations performed in Québec is presented in Supporting Information Figure S1. The Upper St. Lawrence River is represented by pooled samples taken at a network of spawning locations within the Thousand Islands (TIN) where long‐term Muskellunge population monitoring occurs (Farrell et al., 2017; Kapuscinski et al., 2013).

**Table 1:** Genetic diversity parameters

Sampling location	Code	Type	*n*	*H*_o_	*H*_s_	P	Pi	*N*_e_ [95% CI]
Upper Saint Lawrence								
Thousand Islands Network	TIN	Stocked	18	0.154	0.160	0.181	0.0033	362 [150–inf ]
St. Lawrence Lake	LSW	Stocked	15	0.169	0.174	0.177	0.0034	669 [493–823]
Lower Saint Lawrence and inland lake								
Saint‐Louis Lake	LSL	Stocked	55	0.099	0.101	0.305	0.0030	669 [493–823]
Saint‐Françis Lake	LSF	Stocked	61	0.102	0.104	0.286	0.0030	669 [493–823]
Saint‐Pierre Lake	LSP	Stocked	57	0.101	0.102	0.298	0.0030	669 [493–823]
Contrecoeur (St. Lawrence River)	MSC	Stocked	16	0.163	0.168	0.176	0.0034	669 [493–823]
Boucherville (St. Lawrence River)	MSG	Stocked	19	0.144	0.150	0.209	0.0033	669 [493–823]
Varennes (St. Lawrence River)	MSV	Stocked	16	0.161	0.165	0.189	0.0034	669 [493–823]
Tributaries								
des Deux‐Montagnes Lake	LDM	Stocked	56	0.110	0.114	0.287	0.0030	146 [80–298]
De l'Achigan River	ACH	Stocked	25	0.149	0.154	0.170	0.0029	17 [11–178]
Chaudière River	CHD	Introduced	15	0.196	0.198	0.150	0.0032	375 [84–inf]
Maskinongé Lake	MSK	Stocked	25	0.131	0.136	0.236	0.0032	52 [24–145]
Ottawa River	OTT	Stocked	7	0.255	0.277	0.110	0.0035	45 [17–inf]
Yamaska River	YAM	Stocked	16	0.180	0.183	0.137	0.0030	62 [29–129]
Stocking sources								
Chautauqua Lake	CHQ	Source	41	0.129	0.129	0.210	0.0028	137 [95–175]
Pigeon Lake	PIG	Source	27	0.088	0.086	0.152	0.0019	79 [36–178]
Joseph Lake	JOS	Source	30	0.139	0.142	0.190	0.0029	137 [95–175]
Tremblant Lake	TRE	Source	29	0.152	0.157	0.160	0.0029	137 [95–175]
Stocked lake								
Champlain Lake	CHP	Stocked	23	0.165	0.174	0.159	0.0031	14 [6–31]
Frontière Lake	FRO	Introduced	31	0.145	0.148	0.170	0.0029	137 [95–175]
Unstocked lake								
Traverse Lake	TRA	Wild	35	0.070	0.066	0.154	0.0016	NA [NA]
Saint‐Maurice River	MAU	Wild	26	0.172	0.174	0.161	0.0031	45 [25–77]

Notes

Code = acronyms for each sampling site, *n* = number of individuals genotyped, *H*_o_ = observed heterozygosity, *H*_s_ = gene diversity, *P* = proportion of polymorphic loci. Pi = nucleotide diversity (Tajima, 1989), *N*_e_ = effective population size with 95% CI obtained from jackknifing over individuals. Effective population size was computed by merging the major group within the St. Lawrence together. Similarly, all populations from the same stocking group (JOS, TRE, and FRO) were merged in order to increase the sample size. Effective population size could not be estimated for Traverse Lake when implementing a correction for linkage and resulted in an estimated of *N*_e_ = 3 [95% CI = 2–4] without correction.

### 2.2 Molecular methods and SNP calling

A salt‐extraction protocol adapted from Aljanabi and Martinez (1997) was used to extract genomic DNA. Sample quality and concentration were checked on 1% agarose gels and a NanoDrop 2000 spectrophotometer (Thermo Scientific). Concentration of DNA was normalized to 20 ng/µl. Libraries were constructed following a double‐digest RAD (restriction‐site‐associated DNA sequencing; Andrews, Good, Miller, Luikart, & Hohenlohe, 2016) protocol modified from Mascher, Wu, Amand, Stein, and Poland (2013). Genomic DNA was digested with two restriction enzymes (*Pst*I and *Msp*I) by incubating at 37°C for 2 hr followed by enzyme inactivation by incubation at 65°C for 20 min. Sequencing adaptors and a unique individual barcode were ligated to each sample using a ligation master mix including T4 ligase. The ligation reaction was completed at 22°C for 2 hr followed by 65°C for 20 min for enzyme inactivation. Samples were multiplexed (*n* = 48 individuals) to insure fish from each sampling location were sequenced on a minimum of six different multiplexes to avoid pool effects. Libraries were size‐selected using a BluePippin prep (Sage Science), amplified by PCR, and sequenced for 96 individuals on a Ion Proton P1v2 chip that generated approximately 80 million reads per chip. Based on the number of reads for each individually barcoded sample, the prepared DNA was re‐pooled into a new library where the representation of samples with low reads counts was increased. This new library was sequenced on two additional Ion Proton chips to normalize the number of reads per sample.

### 2.3 Bioinformatics

Barcodes were removed, and reads were trimmed to 80 pb using cutadapt (Martin, 2011) allowing for an error rate of 0.2 and demultiplexed using the “process_radtags” module of Stacks v1.44 (Catchen, Hohenlohe, Bassham, Amores, & Cresko, 2013). They were aligned to the Northern Pike (*Esox lucius*) reference genome Eluc_V3 (Rondeau et al., 2014) using bwa‐mem (Li, 2013) with default parameters. Here, 19 randomly distributed individuals with <2.5 million reads were removed, and 643 of 662 Muskellunge were retained for subsequent analysis. Then, aligned reads were processed with Stacks v.1.44 for SNPs calling and genotyping. The “pstacks” module was used with a minimum depth of 3, and up to three mismatches were allowed in catalog assembly. We then ran the “populations” module to produce a vcf file that was filtered with a custom python script. To control for paralogs and HWE disequilibrium, SNPs were retained with a read depth higher than 5, and presence in 70% of each individual at each sampling location and had heterozygosity <0.60. We removed any SNPs that lacked presence in at least 70% of the dataset (max‐missing 0.70 in vcftools) that did not eliminate a variant. The resulting vcf file comprised 16,266 SNPs spread over 11,458 loci and represented the least stringent dataset used to estimate basic diversity parameters. This vcf file was subsampled to meet model assumptions (in particular the use of unlinked SNPs) underlying the population genetic analysis applied. A haplotype file was exported from the “populations” module in Stacks providing information on each RAD locus instead of single SNPs. These loci were then used to estimate a range of statistics described below and to perform FineRADstructure analysis.

### 2.4 Genetic diversity analysis

Hierfstat (Goudet, 2005) was used to compute patterns of observed heterozygosity and gene diversity after excluding monomorphic markers from each sampling location. We then computed the proportion of polymorphic loci in each sampling location using custom R scripts. Contemporary effective population size (*N*_e_) was estimated using the bias‐corrected version based on linkage disequilibrium and assuming random mating (Waples, 2006; Waples & Do, 2008) and implemented in NeEstimator (Do et al., 2014). We kept one SNP per locus and filtered the dataset for physical LD using plink (Chang et al., 2015). Windows of 50 SNPs were shifted by five SNPs each iteration and the routine removed any SNP with a variation inflation factor >2. Nonpolymorphic loci were removed in each population as well as singletons. Effective population size (*N*_e_) was computed after merging sampling locations into major genetic clusters and applying equations from Table 2 of Waples (2006). All burrow estimates for pairs of SNPs located on the same chromosome were removed prior to *N*_e_ estimation. Finally, we estimated nucleotide diversity (Tajima, 1989) and differentiation statistics (*D*_xy_, *D*_a_, *F*_st_) for each RAD locus using mscalc (Ross‐Ibarra, Tenaillon, & Gaut, 2009; Ross‐Ibarra et al., 2008; Roux et al., 2011).

**Table 2:** Simulation results for the 20 ms showing the lowest RMSE

		Mean admixture *q*‐value									Pairwise * F*_ST_			Polym	RMSE
		Admixture with SRC (grp1)			Admixture with LDM (grp2)			Admixture with SLR (grp3)			SLR versus LDM	SLR versus SRC	LDM versus SRC	(1% MAF)	
		LDM	SLR	SRC	LDM	SLR	SRC	LDM	SLR	SRC					
Model	Empirical value	0.101	0.033	0.978	0.881	0.102	0.020	0.018	0.865	0.002	0.036	0.23	0.178	3,619	
	m23_N600_D50	0.070	0.037	1.000	0.916	0.077	0.000	0.015	0.887	0.000	0.012	0.206	0.293	3,619	0.05
	m21_N600_D200	0.112	0.068	1.000	0.863	0.066	0.000	0.026	0.866	0.000	0.012	0.197	0.293	3,621	0.57
	m23_N600_D400	0.063	0.034	0.989	0.904	0.096	0.011	0.032	0.870	0.000	0.012	0.209	0.297	3,616	0.94
	m23_N600_D300	0.057	0.035	1.000	0.929	0.088	0.000	0.013	0.877	0.000	0.013	0.206	0.296	3,615	1.16
	m21_N600_D300	0.116	0.073	1.000	0.875	0.070	0.000	0.010	0.858	0.000	0.012	0.193	0.286	3,615	1.21
	m21_N600_D400	0.106	0.082	1.000	0.866	0.067	0.000	0.028	0.852	0.000	0.012	0.192	0.285	3,624	1.38
	m09_N600_D0	0.116	0.074	1.000	0.867	0.098	0.000	0.017	0.828	0.000	0.011	0.194	0.285	3,625	1.60
	m22_N600_D200	0.069	0.051	1.000	0.918	0.036	0.000	0.013	0.914	0.000	0.013	0.202	0.292	3,629	2.87
	m22_N600_D400	0.076	0.053	1.000	0.910	0.049	0.000	0.012	0.898	0.000	0.012	0.203	0.295	3,630	2.92
	m20_N600_D300	0.057	0.036	0.949	0.797	0.140	0.051	0.146	0.824	0.000	0.005	0.212	0.286	3,604	4.17
	m10_N600_D0	0.087	0.048	0.968	0.844	0.118	0.031	0.068	0.833	0.001	0.013	0.200	0.294	3,638	5.20
	m21_N600_D50	0.137	0.070	1.000	0.853	0.106	0.000	0.011	0.824	0.000	0.012	0.192	0.286	3,641	5.97
	m11_N600_D0	0.058	0.041	0.989	0.905	0.033	0.011	0.037	0.926	0.000	0.013	0.206	0.297	3,597	6.17
	m02_N600_D0	0.000	0.000	0.866	0.712	0.067	0.134	0.288	0.933	0.000	0.007	0.227	0.297	3,596	6.32
	m22_N600_D300	0.073	0.047	1.000	0.911	0.064	0.000	0.016	0.888	0.000	0.013	0.203	0.297	3,596	6.38
	m04_N600_D0	0.027	0.015	0.947	0.802	0.075	0.053	0.170	0.909	0.000	0.005	0.216	0.288	3,594	7.01
	m17_N600_D0	0.034	0.020	0.946	0.762	0.112	0.054	0.204	0.868	0.000	0.006	0.218	0.291	3,592	7.50
	m22_N600_D100	0.078	0.046	1.000	0.875	0.032	0.000	0.047	0.921	0.000	0.012	0.203	0.294	3,647	7.79
	m23_N600_D200	0.066	0.038	1.000	0.923	0.054	0.000	0.011	0.908	0.000	0.014	0.208	0.298	3,650	8.59
	m22_N600_D50	0.075	0.050	1.000	0.913	0.052	0.000	0.011	0.898	0.000	0.013	0.201	0.290	3,654	9.70

Notes

SRC = source population used for stocking. Given the shared patterns of ancestry between the populations used for stocking (i.e., Chautauqua, Joseph, and Tremblant lakes), individuals were merged together and modeled as a single unit. Given that no sign of ancestry from Pigeon Lake was found in the St. Lawrence (SLR) or Deux‐Montagnes L. (LDM), this source was not included here. Based on our empirical results, the migration rate (m) was set 1.5 times higher in LDM than in SLR in which we included LSL, LSF, LSP, MSC, MSG, and MSV. Admixture and *F*_ST_ values in bold are those observed in our empirical data. The group 1 (grp1), group 2 (grp2), and group 3 (grp3) represent admixture membership probability of individuals from LDM, SLR, and SRC, respectively, for *K* = 3. Polym = number of polymorphic sites with a MAF equal to 1% or more. RMSE = root‐mean‐squared error. The lower the RMSE, the closer a given model is to the empirical observation. Here, the 20 best models in terms of RMSE are displayed. Further details can be found in Supporting Information Table S3.

### 2.5 Genetic differentiation and population admixture

Levels of genetic differentiation between sampling locations were computed using Weir and Cockerham's *F*_st_ estimator *θ* (Weir & Cockerham, 1984) in vcftools v0.1.15, and resulting values were used to construct a heatmap and a dendrogram in R using custom script. Confidence intervals around *F*_st_ were computed using Hierfstat with 1,000 bootstraps. Isolation by distance (IBD) was tested between pairwise genetic distances as *F*_st_/(1 − *F*_st_) (Rousset, 1997) and the pairwise distance following the river watershed network using a Mantel test in R with 10,000 permutations.

To better understand whether low genetic differentiation was due to high population connectivity, we also measured the level of migration within two sites at the extreme ends of the St. Lawrence mainstem sampling using an approach similar to Migrate (Beerli & Felsenstein, 2001). We performed coalescent simulations of two populations of independent size *N*_1_ and *N*_2_, connected by migration (*M* = 4*N*_ref_ *m*), modeled independently, and therefore with possible asymmetry. We modified an ABC pipeline (described in Supplementary Materials: Appendix S1) and estimated the intensity of gene flow between the sites with conventional ABC procedures (Csilléry, Blum, Gaggiotti, & François, 2010).

Our simulation pipeline was also applied to examine whether strong genetic differentiation (as measured by *F*_st_) translated into a long divergence time. We estimated the divergence time between Chautauqua and Pigeon lakes, the two original sources of stocking, under the assumption that no gene flow existed between these contemporary isolated lakes. Under this hypothesis, the divergence history can be summarized by a model of strict isolation (SI) characterized by the effective population size of the two daughter populations (N1 and N2) that diverged from a common ancestral population of size Nanc at time Tsplit. Coalescent simulations were again used to generate genomic data and to compare these theoretical patterns to our empirical data using an ABC framework. Full details are provided in Supplementary Materials: Appendix S1. This analysis was replicated five times by comparing a randomly chosen site within the St. Lawrence to either Chautauqua or Pigeon Lake.

Next, ancestry and admixture proportions were inferred for each individual using Admixture (Alexander et al., 2009) with *K*‐values ranging from one to 25. Then the snmf function implemented in the LEA R package (Frichot & François, 2015) to estimate ancestry coefficients levels of *K*. Other DAPC model‐based clustering methods (Jombart, Devillard, & Balloux, 2010) produced highly congruent results to those obtained using Admixture or LEA. All admixture analyses were performed by maintaining a single SNP per locus. Keeping either a random SNP or the SNP with the highest minor allele frequency produced similar results (not shown), and we report only the results of analyses performed with SNPs showing the highest minor allelic frequency (MAF) at each locus. We then correlated the individuals “stocking source” ancestry coefficient to individual levels of observed heterozygosity (Spearman's rho) and tested it for significance with a *t* test.

Finally, we used a modification of the fineSTRUCTURE package (Lawson, Hellenthal, Myers, & Falush, 2012) implemented in FineRADstructure (Malinsky, Trucchi, Lawson, & Falush, 2018) to infer levels of population genetic structure and ancestry from haplotype data derived from RAD loci. Less than one percent of missing data were allowed. First, RADpainter was used to compute the co‐ancestry matrix. Then individuals were assigned to populations with fineSTRUCTURE using 100,000 MCMC iteration for burn‐in and the same number of sampled iteration with a thinning interval of 1,000.

### 2.6 Demographic simulations of historical stocking

Individual forward simulations were used to investigate the effect of stocking on admixture patterns and the contemporary genetic makeup of populations in the fluvial system of the Lower St. Lawrence River (a series of connected lakes with the mainstem). Tributaries were not simulated as data on their stocking intensity were not consistently available. The goal of the simulations was to estimate the effective migration rate from stocking sources to the St. Lawrence population necessary to explain observed admixture and benefit our understanding of stocking effects (i.e., levels of introgression). We focused on a restricted dataset to construct a simplified model of divergence made of three Muskellunge groupings along the Lower St. Lawrence fluvial system. The first group included fish from sites within the fluvial lakes and the mainstem separating lakes within the St. Lawrence River, namely lakes Saint‐Pierre (LSP), Saint‐Françis (LSF), and Saint‐Louis (LSL), as well as from three different sampling mainstem localities between Montréal and Sorel including Contrecoeur (MSC), Boucherville (MSG), and Varennes (MSV) sampling sites (*n = *224 individuals, named SLR hereafter). A second population (lake des Deux‐Montagnes, LDM *n = *56) was identified based on patterns of admixture and shared ancestry (see Section 3). Finally, a third population was the source of stocking populations and included as a single group the original site CHQ as well as JOS and TRE lakes (*n = *100) that originated from stocking with CHQ (confirmed by admixture and FineRADstructure analysis, see Section 3). Finally, the other source of stocking, PIG from the Kawartha system was not considered since no contribution to the admixture patterns in the St. Lawrence River was detected (see Section 3).

We simulated a neutral 10,000 kb long chromosome assuming a uniform mutation rate *µ* of 1e‐8 per bp per generation and a recombination rate *r* of 1e‐8 per bp per generation using slim v2.6 (Haller & Messer, 2009). First, we simulated an ancestral ideal population of size *N* = 2,400 for 80,000 generations to reach equilibrium. The ancestral population was then split into three populations corresponding to the stocking source (SRC), the LDM, and the SLR with a reduced initial size of *N* = 800 (a range of different sizes were tested, see below). Given observed data, we allowed for a constant rate of migration between LDM and SLR with *m*_SLR‐>LDM_ = 0.0005 and *m*_LDM_→_SLR_ = 0.00025 (we explored parameters producing data similar to empirical observations). We implemented asymmetric dispersal to reflect the expected downstream biased dispersal (e.g., Paz‐Vinas, Loot, Stevens, & Blanchet, 2015), which was also observed in our data from LDM into SLR (see admixture results). Initially, no migration was allowed between the stocking sources (considered as a single unit based on patterns of shared ancestry) and either LDM or SLR. These populations kept diverging for 2,685 generations roughly corresponding to the postglacial divergence period (assuming a generation time of 6 years based on age at first spawning; Farrell et al., 2007). To reproduce initial stocking event about 15 generations ago, we introduced migration between stocking sources and LDM and SLR. Since more admixture was observed in LDM compared to SLR (see Section 3), migration into LDM was set 1.5 times higher than in SLR. We tested a range of migration rate from 1e‐6 to 0.1. After 82 685 generations, a set of individuals matching our empirical sample sizes were randomly sampled and exported into vcf format for computation of summary statistics corresponding to admixture levels, Weir & Cockerham *F*_ST_ and the numbers of polymorphic site above the 1% MAF. We allowed for lower fitness of the stocking source population by implementing a mortality‐based filter (fitness callback in Slim v2, Haller & Messer, 2017). We explored a set of fitness filters where 50, 100, 200, and 400 randomly chosen stocked individuals died, implying a mortality rate of 6%, 12.5%, 25% and 50% for *N* = 800. This allowed testing outcomes if a small number of individuals reproduced, while additionally considering effects of a variety of mortality‐based filters such as lower local adaptation of migrant individuals. Finally, we tested the effect of varying demographic scenarios through population increase or decrease by multiplying the size of each descendant populations by 2 (*N* = 1,600), 0.75 (*N* = 600, closer to the estimated effective population size) and 0.5 (*N* = 400). The ancestral population size was multiplied accordingly. The number of individuals that died was kept constant, implying a varying mortality rate. Simulations were repeated 50 times to assess the variability of admixture inferences. We then computed the root‐mean‐squared error (RMSE) for each scenario and computed the distance between the distributions of summary statistics and the empirically observed one, allowing us to compare model fit to the data. Data could have been analyzed in an Approximate Bayesian Computation framework (Beaumont, Zhang, & Balding, 2002) to test realistic scenarios of migration and subsequent admixture. However, the forward simulations implemented were too slow to generate the required number of simulations (i.e., >500,000) for classical ABC analysis. All scripts used with Slim will be freely available Github at https://github.com/QuentinRougemont/fwd_sims

## 3 RESULTS

### 3.1 Genetic diversity

An average of 3.23 million reads per individual was sequenced. Genetic diversity indices measured on each rad loci revealed a median п value of 0.003. Median observed heterozygosity and gene diversity for polymorphic SNPs markers were 0.144 and 0.148, respectively (Table 1). Two sites stood out as displaying significantly lower observed heterozygosity compared with other sites, namely the populations from TRA (*H*_o_ = 0.088, *H*_s_ = 0.086, *Wilcoxon‐test*, *W* = 759 *p* < 0.001) and PIG (*H*_o_ = 0.070, *H*_s_ = 0.066, *Wilcoxon‐test*, *W* = 330, *p* < 0.001). Lower diversity for these populations was reflected by their п estimates of 0.0016 and 0.0019, respectively (Table 1). Lakes of the St. Lawrence system (des Deux‐Montagnes [LDM], Saint–Louis [LSL], Saint‐Françis [LSF], and Saint‐Pierre [LSP] Lakes) also displayed slightly lower diversity than median diversity values (median *H*_o_ = 0.103, *H*_s_ = 0.105) whereas the opposite was observed for sites located in the fluvial stretch of the St. Lawrence between LSL and LSP (MSG, MSC, and MSV; median *H*_o_ = 0.156, *H*_s_ = 0.161). The median proportion of polymorphic SNPs was 18% (*SD* = 6%), with the least polymorphic site being the Ottawa River (OTT; 11%) as expected given its small sample size (*n* = 7), whereas LSL was the most polymorphic location (31%). On average, there was more polymorphism within the St. Lawrence than in the tributaries or lakes (Table 1). There was 21 times more private polymorphisms into the St. Lawrence mainstem compared to those within tributaries. The St. Lawrence mainstem also contained 2.7 times more private polymorphism than all stocking sources merged together. Additionally, on average, there were 10 times more private polymorphisms in the stocking source when compared to tributaries and adjacent lakes. *N*_e_ estimates returned low values ranging from 14 (CHP) to 2,308 (LSP) with four cases where the *N*_e_ value could not be estimated, either because of paucity of polymorphic markers and/or limited sample size such as for OTT (result not shown). Pooling all Muskellunge according to their inferred genetic cluster (see below) resolved this problem and revealed for example a *N*_e_ value of 669 (95% CI = 493–823) for the St. Lawrence group (Table 1). Without correction for physical linkage all *N*_e_ values were downwardly biased, corroborating findings by Waples, Larson, and Wasples (2016).

### 3.2 Population differentiation

**Figure 2:**
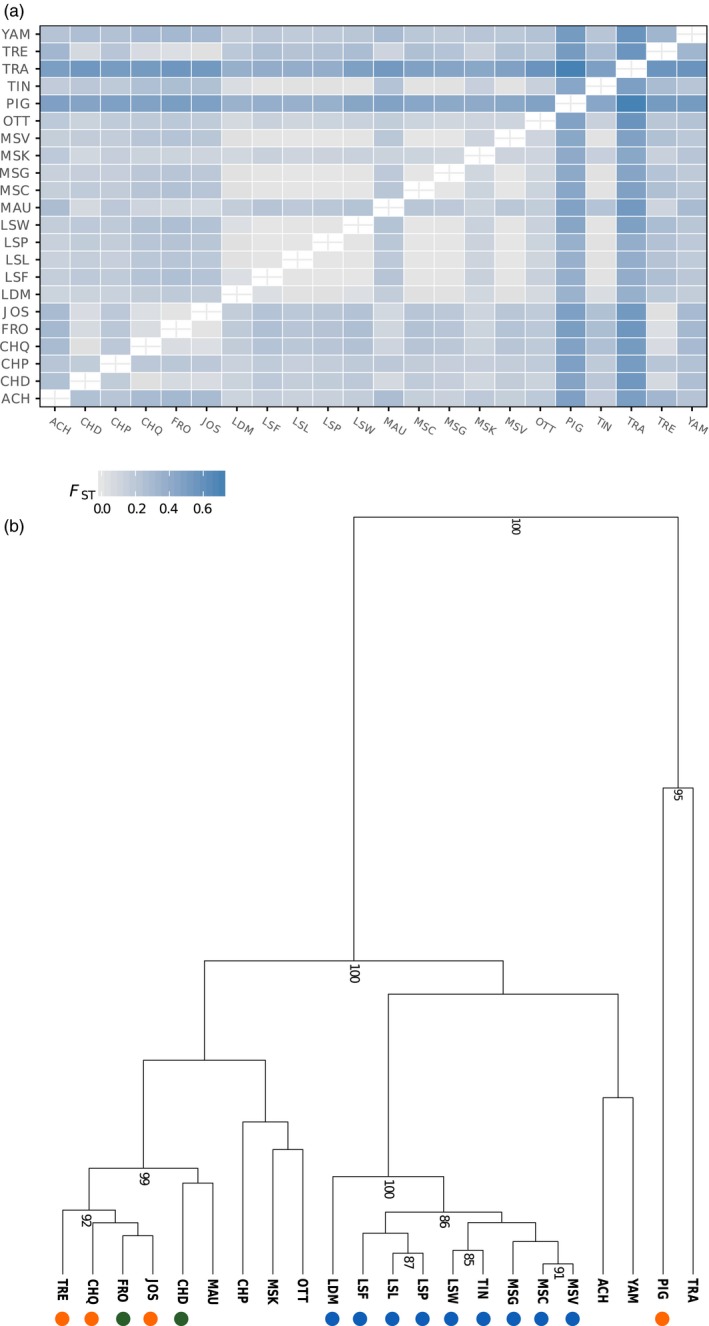
(a) Heatmap of pairwise *F*_ST_ values between each sample site. (b) Clustering based on pairwise *F*_ST_ values performed on each sample site. Only nodes with a bootstrap support higher than 80 are displayed. Orange dots = source of individuals used for stocking. Green dots = lake or river where Muskellunge were absent and have been introduced. Blue dots = site from the St. Lawrence River and des Deux‐Montagnes Lake

The global *F*_ST_ estimate averaged over‐all pairwise comparisons, was 0.211 (ranging from 0.0006 between LSL and MSC to 0.709 between TRA and PIG). Five locations from the Lower St. Lawrence River (i.e., LSL vs. MSG, LSL vs. MSC, MSG vs. MSC and both MSC and MSG vs. MSV) had nonsignificant pairwise *F*_ST_ values (Figure 2a; Supporting Information Table S1). When excluding all tributaries and inland lakes, *F*_ST_ values averaged over all sites from the Thousand Islands upper river network of spawning sites (TIN) in the Upper St. Lawrence River downstream to LSP was 0.014 (Figure 2a). Here, the LDM site clustered separately from all sites located on the Upper and Lower St. Lawrence (Figure 2b). When removing the LDM site, the *F*_ST _among all other sampling locations dropped to 0.008, suggesting weak genetic differentiation throughout the mainstem of the St. Lawrence River. LSL, LSP, LSF, and LSW displayed significant but weak *F*_ST_ (i.e., <0.009) between each other and between the three sites from the St. Lawrence section including MSC, MSG, and MSV. The uppermost St. Lawrence population (TIN) displayed higher differentiation when compared to all other sites with *F*_ST_ ranging from 0.015 to 0.055.

All tributaries were significantly differentiated from the St. Lawrence R. sites between LSL and LSP and displayed a much stronger level of differentiation than observed within the St. Lawrence R. itself, with an averaged *F*_ST_ value between each tributary and all sites from the St. Lawrence R. of 0.20 for the Saint‐Maurice R. (MAU), 0.119 for OTT, 0.177 for the Chaudière R. (CHD), and 0.196 for the Yamaska R. (YAM). With population tree topology, the CHD and MAU populations, both with presumably historically low abundance that were subsequently stocked, clustered close to the stocking source, yet their distinctiveness was strongly supported (Figure 2b). Differentiation among Maskinongé L. (MSK), Ottawa R. (OTT) and Champlain Lake (CHP) was less clear as they appeared close to the source of stocking albeit the clustering was weakly supported in the tree (Figure 2b). There was a modest signal of IBD when all sites were included (mantel test *r* = 0.369 *p* = 0.0129) and considering only St. Lawrence R. sites (from TIN to LSP) a stronger IBD pattern was revealed (*r* = 0.799, *p* = 0.0046, Figure 2). Finally, pairwise *D*_xy_ and net divergence (*D*_a_) did not revealed major difference between compared pairs of populations (Supporting Information Table S2).

### 3.3 Evidence for asymmetric gene flow

ABC estimates of migration rates and effective population size were performed using 1 million coalescent simulations. We chose the site LSP and LSP, located at the opposite ends on the Lower St. Lawrence and did not use the more upstream site (e.g., TIN) located upstream of dams artificially restricting upstream migration. Results indicated that posterior distribution of the migration rate was highly different from the prior distribution, resulting in narrow credible intervals (Supporting Information Figure S5). This migration was strongly asymmetric with a median value *M*_down←up_ = 40 [95% credible intervals = 39–40] whereas migration from downstream to the upstream site was low with a median value *M*_down←up_ = 0.067 [95% credible intervals = 0.018–0.140]. Effective population size was similar between downstream and upstream sites (Supporting Information Figure S5) This provided support for the hypothesis that low genetic differentiation was partly due to strong downstream directed dispersal. Relatively large effective population size may also contribute to this pattern (Supporting Information Figures S5 and S6).

### 3.4 Evidence for a relatively recent and shared history of divergence

Estimates of time since divergence were performed assuming that the divergence history could be summarized by a simplified model of SI (Supplementary results: Appendix S1). Posterior distribution of the divergence time between the two major sources of stocking were differentiated from the prior distribution, indicating that this parameter could confidently be interpreted. We found, that the median value was 0.023 coalescent units [95% CI = 0.010–0.045]. Assuming a mutation rate *µ* of 1*e* ^−8^ bp per generation (see Supplementary results: Appendix S1 for treatment) and the 5 years generation time, this would translate to a divergence time of 23,000 years [10,000‐45,000]. Replicating this analysis using other sites (Supplementary results: Appendix S1, Supporting Information Figures S7–S17) resulted in lower estimates of divergence time (i.e., median range from 15,400 to 19,300 years, min = 6,500, max = 31,000).

### 3.5 Population structure and admixture

**Figure 3:**
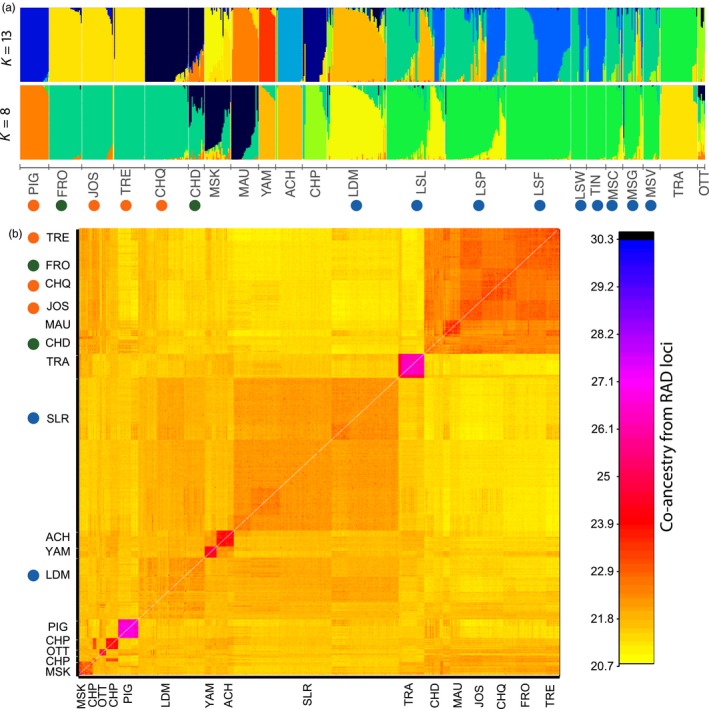
(a) Levels of admixture for *K* = 8 and *K* = 13. Each color represents a distinct cluster, and each bar represents an individual. See Figure 1 for the labels of each dot in the graph; (b) co‐ancestry matrix inferred by FineRADstructure. Each pixel represents individual co‐ancestry coefficient inferred based on short haplotype loci. The labels summarize the major groups according to names of sampling sites. The higher values of co‐ancestry coefficient sharing are depicted in darker colors, whereas lower values of co‐ancestry coefficient sharing appear in yellow colors

Analyses of admixture cross‐validation consistently produced low cross‐validation scores for *K* = 8 and *K* = 13 indicating the number of clusters likely lies between these values (Supporting Information Figure S3). Similar results were obtained from LEA cross‐entropy criterion with minimum values obtained around *K* = 12 to 13 while the DAPC provided the same results. For simplicity, only results from the admixture analysis for *K* = 8 and *K* = 13 are detailed below (see also Figure 3a). Further plots are presented in Supporting Information Figure S3 for additional *K* values (up to *K* = 19).

Considering *K* = 8 clustered together the major groups used for stocking (i.e., CHQ, JOS, TRE) together with the FRO introduced from the JOS population (mean *q*‐value for the four lakes = 0.975; Figure 3). Below, we considered that any individual from the St. Lawrence and lake who displayed a stocked ancestry coefficient above 0.10 to be admixed, as commonly done in the literature (e.g., Valiquette, Perrier, Thibault, & Bernatchez, 2014, Létourneau et al., 2018). We defined “stocked ancestry” as the group FRO‐JOS‐TRE‐CHQ and the group PIG.

Based on this criterion, we observed variable levels of admixture in all tributaries and lakes outside the mainstem of the St. Lawrence River. From MAU, 13 fish (50%) displayed a stocking source membership probability >0.10. Specifically, one individual was assigned to the “stocking” group with a *q*‐value >0.95 and remaining individuals displayed mixed membership probability suggesting backcrosses or presence of advanced generation hybrids (*q*‐values between 0.116 and 0.626). The remaining individuals (50%) from MAU displayed a *q*‐value >0.90 belonging to another group comprised of individuals from MAU, CHD and MSK. All 15 Muskellunge from CHD displayed mixed ancestry with membership probability of belonging to the “stocking” group ranging from 0.407 to 0.724 and individuals from MSK were admixed (*q*‐values ranging from 0.22 to 0.88). Among those, nine (36%) displayed *q*‐value of belonging to the “stocking group” ranging from 0.10 to 0.617 with no individual of apparent pure “stocking” origin (Figure 3a). Six (37.5%) and four individuals (57%), respectively, from YAM and OTT were admixed (*q*‐value > 0.10) with the stocking source (Figure 3a). None of these individuals was of pure stocking origin as they displayed *q*‐values ranging from 0.10 to 0.211 and from 0.10 to 0.365 for YAM and OTT, respectively. In OTT, all seven sampled Muskellunge were admixed and none was assigned to a particular cluster. The Achigan R. (ACH) and YAM populations clustered together (mean *q*‐value = 0.915) whereas TRA and CHP populations formed two separate clusters (*q*‐value = 0.96 and 0.81, respectively). Finally, the Champlain L. showed indicators of significant admixture from the stocking source for six individuals (30%). There were 2 F_0_ immigrants and 5 introgressed individuals with *q*‐values ranging from 0.10 to 0.525.

Sites from the St. Lawrence River from the uppermost (TIN) to the most downstream site (LSP) tended to form a single group with the exception of LDM that clearly clustered separately (*q*‐value = 0.84). In contrast with inland lakes and tributaries, weak evidence was detected for admixture with the stocking source population. Thus, 21 individuals (8%) from the St. Lawrence R. (from TIN to LSP) displayed introgression with the stocking source (*q*‐value ranging from 0.10 to 0.54) and no individual was of pure stocking origin. However, we observed more fish (*n* = 19, 33%) from LDM that were admixed with the stocking source (*q*‐value ranging from 0.10 to 0.50) but none were of pure stocking origin. While there was limited admixture with the stocking source, we found evidence for admixture among fish from different locations within the system. Here, 6% of individuals from LSL were classified as F0 immigrants (*q*‐value > 0.9) from the nearby LDM population and 42% were admixed with LDM (Figure 3a). It is noteworthy that considering the spatial location of individuals in LSL (all collected fish within the SLR were georeferenced by GPS), we observed a tendency for the most admixed individuals (78%) to be preferentially found in the northern part of LSL whereas 87% of pure individuals were located preferentially in the southern part of LSL. Finally, 17 Muskellunge (30%) from LSP displayed admixture with LDM whereas 11 individuals (22%) from the St. Lawrence R. including MSG, MSC, and MSV were admixed with LDM (*q*‐value>0.10).

**Figure 4:**
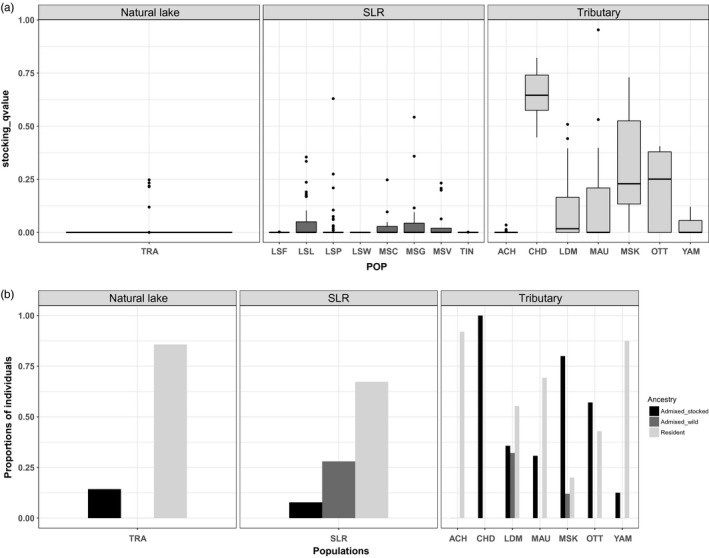
(a) Boxplot of “stocking source” ancestry coefficient (*q*‐values) in the single nonsupplemented population and in the different supplemented populations separated according to whether they occur in the St. Lawrence River itself (SLR) or its tributaries. (b) Proportion of individuals assigned to different classes of ancestry: individual assigned as “resident” if *q*‐value >0.9, individual assigned as of “admixed domestic ancestry” if *q*‐value of belonging to a “stocking” source cluster was above 0.1, individual assigned as of “wild ancestry” if *q*‐value of belonging to another foreign population was above 0.10.

Considering *K* = 13 revealed the same general pattern of differential admixture and weak admixture with the stocking source was observed at all sites within the mainstem of the St. Lawrence R., a somewhat more pronounced admixture in LDM and the highest admixture being observed in tributaries and inland lakes (except FRO where no Muskellunge occurred before stocking) (Figure 3a). However, *K* = 13 revealed more separation between some population clusters. Thus, Muskellunge from YAM and ACH tributaries were clearly assigned to two clusters corresponding to their local river. The original stocking source (CHQ) clustered independently (mean *q*‐value = 0.95) of derived individuals of JOS‐TRE stocking sources that grouped together (*q*‐value = 0.96). While FRO lake still clustered with JOS, fish from MAU clustered independently (*q*‐value = 0.87) but with one individual showing a F_0_ immigrant profile (*q*‐value > 0.95) from the JOS‐TRE stocking group and seven other individuals showing signs of introgression (0.10 < *q*‐value < 0.53) (Figure 4a,b). Five Muskellunge (20%) from MSK displayed a *q*‐value > 0.90, while 95% of remaining individuals showed introgression (0.13 < *q*‐value < 0.730) with the JOS‐TRE stocking source (Figure 3a, Figure 4). The CHP site tended to form a separate cluster (mean *q*‐value = 0.81) but with one F_0_ immigrant from CHQ stocking source and five individuals (21%) showing admixture (F1 like profile) from the JOS‐TRE group (Figure 3a). Individuals from the St. Lawrence River from TIN to LSP were now separated into two admixed groups. In particular, individuals from the uppermost TIN site were assigned to this cluster (mean *q*‐value = 0.87) while 31%, 26%, 12%, and 7% of individual, respectively, from LSF, LSW, LSL, and LSP were now assigned (*q* > 0.90) to this new group. In these same sites, respectively, 31%, 27%, 22%, and 37% of individuals, as well as 3.5% in LDM were admixed (Figure 3a, top panel). Finally, five individuals (9%) from LSL were assigned as putative F_0_ immigrants from LDM and 16 individuals (29%) displayed mixed membership probability. The same was observed in LSP with no F_0_ immigrants but 15 individuals (26%) admixed with LDM. Overall, Muskellunge from the St. Lawrence displayed lower stocking *q*‐value membership than the LDM or tributaries and inlands lakes (SLR range: 0.10 to 0.63, LDM range: 0.10 to 0.51, tributaries range: 0.10 to 0.99; see details and boxplots in Figure 4a). Similarly, the number of admixed individuals was lower in the St. Lawrence (8%) than in the LDM (31%) and tributaries and inland lakes (42%) (Figure 4b). Finally, there was a global significant and positive correlation between the level of stocking ancestry and the observed heterozygosity for *K* = 13 (rho spearman = 0.37, *t* = −15.578, *df* = 452.03, *p*‐value < 2.2e‐16) as well as for *K* = 8 (rho spearman = 0.26, *t* = 12.863, *df* = 513.38, *p*‐value < 2.2e‐16).

Considering higher *K* values (from 14 to 16) resolved all rivers except the CHD (that was admixed with CHQ and LDM), only a value of *K* = 19 separated CHD individuals to their own cluster (median‐*q* > 0.90). The FRO and JOS lake remained clustered together (see Supporting Information Figure S1).

Results from co‐ancestry analyses at the haplotype level provided additional information about ancestry between individuals and revealed patterns consistent with admixture analyses at different *K* values and highlight the similarity observed at *K* = 8 and the finer differences for *K* = 13. In particular, two major blocks stood out corresponding to (a) the populations used for stocking and (b) the St. Lawrence R. Here, the CHQ, JOS, and TRE sources as well as the derived populations FRO and CHD appeared closely related (top panel in Figure 3b). All individuals from MAU, 24% from MSK and 9% from CHP shared ancestry with these source populations (Figure 3b). The second major block was made of individuals from the different sampling sites from the St. Lawrence River (TIN to LSP excluding tributaries) and displayed moderately shared co‐ancestry with LDM. In particular, 36% of LSL, 12% of LSP, and 16% of the MSC, MSG, and MSV group of individuals displayed closer co‐ancestry to LDM than to remaining individuals from SLR. Admixture analyses revealed one individual from JOS that displayed close ancestry to the St. Lawrence River fish. The seven individuals from OTT were closely related to a group of five individuals (9%) from LDM, indicating possible downstream dispersal from the Ottawa River to LDM or a common ancestral origin. All fish from ACH and YAM were well separated (*K* = 13 in admixture), but displayed shared ancestry, highlighting their close relationship (*K* = 8 in admixture). Finally, both PIG and TRA individuals formed well‐separated clusters of ancestry (Figure 3b).

### 3.6 Simulations of admixture

Results indicated that over all scenarios, *N* = 600 (closer to estimated *N*_e_ in the SLR) systematically displayed lower RMSE (root mean squared errors) than those with higher *N*_e_ (Supporting Information Table S4). For instance, only *N* = 600 resulted in a similar level of polymorphism (i.e., polymorphic SNPs at the 1% level) as to empirical data, while all other datasets were significantly different in number of polymorphic sites. Therefore, for simplicity, we present results for the *N* = 600 scenario (the 20 best model related to *N* = 600 are presented in Table 2); other scenarios for models with the lowest RMSE are presented in Supporting Information Table S3). The best scenarios in terms of RMSE were those where migration rate from the stocking source (SRC) to the St. Lawrence (LSW to LSP) ranged between *m* = 0.0025 and *m* = 0.005. Migration rate was systematically higher from SRC to LDM with values ranging from *m* = 0.00375 to *m* = 0.0075 (ms M20–M23, similar to M9–M11 but includes mortality filters; Table 2). None of the scenarios where migration rate was set higher (from *m* = 0.01 to *m* = 0.30) provided a good fit (Table 2, Supporting Information Table S3). In particular, M16 theoretically would reproduce most closely the expected level of migration (m ~ 0.5) due to the intensity of stocking, but poorly fitted the data (Supporting Information Table S3). Scenarios M1–M8, with lower migration rates (*m* < 0.001), did not properly reproduce observed data. While the level of genetic differentiation between SRC and SLR or between SRC and LDM were qualitatively close to that observed empirically in models M20–M23 (simulated *F*_ST_ range ~0.192 to 0.298, Table 2), none of the scenarios were able to reproduce the observed divergence between SLR and LDM (mean *F*_ST_ for the 20 best scenarios = 0.011 vs. 0.036 in our data set; Table 2). The difference between simulated and observed differentiation was significant (*p* < 0.005), an indication that some processes not considered in the model, such as a long isolation period or a smaller *N*_e_ in LDM or that two populations originating from evolutionary divergent lineages, may have contributed to their divergence. Overall, scenarios with the mortality‐based filter produced the lowest RMSE, with the best scenario (RMSE = 0.05) where 50 individuals randomly died (Table 2). However, scenario M9 where no individual died also produced a low RMSE of 1.60. Several scenarios with mortality of 100–400 individuals presented intermediate RMSE values (Table 2), indicating that varying rates of mortality for *N* = 600 could explain empirical data. Increasing mortality rate to 500 individuals (i.e., 83%) resulted in RMSE of 1.73.

## 4 DISCUSSION

### 4.1 Genetic structure between distant lakes and isolation by distance within the St. Lawrence River

A common issue in population genetics is delineation of population structure confounded with isolation by distance (IBD), where genetic differentiation increases with geographic distance due to genetic drift under limited dispersal (Wright, 1943). Here, our data indicated both the occurrence of clusters of genetically differentiated groups (i.e., among distant and isolated water bodies) and significant isolation by distance within the St. Lawrence River, as reported in Muskellunge (Kapuscinski et al., 2013; Miller et al., 2017) and other species (Meirmans, 2012; Sexton et al., 2014). The behavior of the Muskellunge, especially its known spawning site fidelity (Farrell et al., 2007) and possible natal homing (Crossman, 1990; Jennings et al., 2011; Margenau, 1994; Miller et al., 2001) are factors that can contribute to both IBD and the establishment of population structure. Regarding the major clusters, a strict interpretation of *F*_ST_ values and admixture results indicates that each lake and each tributary could be classified as a distinct population (Supporting Information Figure S3). However, finer analyses revealed shared co‐ancestry between many samples, especially between some stocked tributaries/lake with population stocking source. Moreover, coalescent analyses suggested a recent divergence of studied populations, and there is evidence for ample shared polymorphism between all populations. We therefore hypothesize that tributaries represent a bottlenecked subset of the larger St. Lawrence R. populations following their colonization by a small number of founders. Then, isolation of each individual group enhanced the effect of genetic drift (e.g., Turnquist et al., 2017), resulting in pronounced population‐level genetic differentiation. In the case of the Chaudière River, we further note that the connectivity with the St. Lawrence is already restricted by natural waterfall. In this particular case, the river display stronger footprint of introgression with individuals of stocking origin.”

The two clusters observed within the St. Lawrence R. are likely due to isolation by distance confounding the clustering methods (Bradburd, Coop, & Ralph, 2018; Meirmans, 2012). As expected, our coalescent analysis supported downstream biased dispersal, which likely explains the high connectivity within the St. Lawrence R. (Morrissey & de Kerckhove, 2009). Moreover, the presence of two dams on the St. Lawrence certainly contributes to restrict upstream dispersal and associated gene flow.

In tributaries, a variable proportion of fish from sites between mainstem LSL and LSP displayed shared ancestry with fish from the LDM tributary. Admixed individuals were preferentially located in the northern shore of LSL, whereas those on the southern shore where generally not admixed. Interestingly, this dichotomy between the northern and southern shores of LSL was also reported in the Northern Pike (Ouellet‐Cauchon, Mingelbier, Lecomte, & Bernatchez, 2014) and Yellow Perch *Perca flavescens *(Leclerc, Mailhot, Mingelbier, & Bernatchez, 2008). In both species, fish from the south shore of the LSL were more similar to those from elsewhere within the St. Lawrence R. than those from the north shore. LSL is characterized by contrasted water masses flowing from the St. Lawrence R. on the south shore and from the Ottawa River on the north shore (Hudon & Carignan, 2008; Leclerc et al., 2008). Therefore, it would be of interest to distinguish the respective contribution of ecological (e.g., temperature, pH, light extinction, etc.) and historical (e.g., glacial refugia) factors responsible for this repeated pattern of divergence observed between the north and south shores of LSL among different species.

### 4.2 Genetic impacts of stocking vary with supplementation history and population size

Correlation between stocking intensity and admixture have previously been documented in a range of species including mainly salmonids (Campos, Posada, & Morán, 2008; Finnegan & Stevens, 2008; Garcia‐Marin, Sanz, & Pla, 1999; Marie, Bernatchez, & Garant, 2010; Perrier, Baglinière et al., 2013; Perrier, Guyomard et al., 2013; Sønstebø, Borgstrøm, & Heun, 2008) Studies additionally revealed a tendency toward a decreasing proportion of admixture following stocking cessation (Hansen et al., 2009; Harbicht, Wilson, & Fraser, 2014; Létourneau et al., 2018; Perrier, Baglinière et al., 2013; Valiquette et al., 2014). Our results are congruent with these observations where PIG lake, initially used as a stocking source (from 1950 to 1965), left few traces of admixture in our populations. Significant “PIG” ancestry was detected in <2% of the individuals. Miller et al. (2009) documented a similar pattern with microsatellite DNA markers following introduction of three sources of Muskellunge including intrabasin transfer of progeny from Shoepack Lake Minnesota. The stocking resulted in admixture from each source and the deleterious effect of slow growth was detected in fish with Shoepack ancestry. Managers responded by altering stocking policies and the strain mixing has since declined over time via dilution (Miller et al., 2012). We hypothesize a similar dilution of individual ancestry through time has occurred with respect to the PIG stocking source although we are aware of no negative effects of its introduction.

Miller et al. (2012) compared genetic mixing following Muskellunge stocking of four strains released in supplemented and introduced populations that showed ancestry from each of the source populations, but also indicated that the quantity of stocking was unrelated to levels of admixture. Our results also indicated that the most extensively stocked populations (Lower SLR) displayed the lowest amount of stocking ancestry. This suggests that since the cessation of stocking in 1998, the majority of chromosomal blocks introgressed from the stocking source populations have been removed from the genome of local fish. In contrast, other supplemented tributaries and lakes (e.g., MSK, LDM, OTT) clearly displayed signs of shared ancestry with the stocking sources. Also, the MAU River, which has no recorded stocking to our knowledge, clustered with the stocking sources suggesting the possible occurrence of migrants from further upstream stocked waters in this watershed. Scribner et al. (2015) similarly demonstrated spread of source populations in Michigan watersheds following stocking as evidence of gene flow from stocked individuals.

The observation of low admixture in the St. Lawrence R. may be expected if the St. Lawrence exhibit a larger population size than currently estimated. Regarding *N*_e_ *, *the estimated size might be downwardly biased due to the subdivided nature of the river and its tributaries and due to admixture (Waples & England, 2011). Moreover, coalescent estimates of *N*_e_ (supplementary analyses) may be more relevant (Sjodin, 2005) but are different from estimates based on LD, highlighting classical difficulties in estimating and interpreting this parameter.

With these difficulties in mind, a first scenario to explain the lower admixture of the St. Lawrence R. involves a purely neutral admixture because of high connectivity (and putative high *N*_e_) in this system. Therefore, it is possible that the footprints of admixture between stocked fish (of apparently lower effective size) would quickly be diluted into the larger population of the St. Lawrence R. For instance, if stocked individuals are only able to hybridize with wild individuals, then the resulting F1 would mate with wild individuals. This would lead to a decline of the “stocked” genome as 1/2^g^ generation (Phillips & Baird, 2015). This could quickly result in low levels of admixture that may not be detectable with global ancestry inference approaches. On the other hand, in the smaller stream populations with putative low effective population size, footprints of admixture are still apparent. In these small populations, stocking could potentially have some positive assets (Frankham, 2015). Given that all populations are likely postglacially derived, they are likely to be genetically compatible with small probability of outbreeding depression (Ralls et al., 2018). Therefore, stocking from slightly diverged populations may contribute to the maintenance of their genetic diversity and evolutionary potential (Carlson et al., 2014; Frankham, 2015).

A second demographic scenario fitting our empirical data involved differences in effective population size, but requires the action of selection. For instance, stocked individuals may display low survival, low reproductive success (hence resulting in little introgression), and/or selection could act against introgression, as supported by several of our models. Under this second scenario, the higher effective population size and pronounced connectivity of the Lower St. Lawrence R. may play a critical role in selection against introgression (Glémin, 2003). Alternatively, genetic drift may overcome selection against introgression in smaller, isolated populations (Frankham, Ballou, & Briscoe, 2010) leading to fixation of “foreign” alleles and higher footprints of admixture. Such variable outcomes of stocking on admixture have been documented in salmonids (Perrier, Guyomard et al., 2013). Low survival and/or reproductive success of stocked fish has been reported mostly in salmonid species characterized by putatively high rate of local adaptation (Araki, Cooper, & Blouin, 2007; Araki et al., 2009; Araki & Schmid, 2010; Christie, Ford, & Blouin, 2014; Thériault, Moyer, & Banks, 2010; Thériault, Moyer, Jackson, Blouin, & Banks, 2011). Similarly, in Muskellunge, low levels of admixture from the same Ohio strain used in our study, was observed following intensive stocking into West Virginia streams despite their high use (White, Kohli, & Converse, 2018). Our results do not necessarily invoke a mechanism of negative selection or lower fitness of individuals to explain observed pattern. Indeed some of our neutral simulations, with modest levels of migration and no mortality, also provided a good fit to the data.

Despite its ease of implementation, to the best of our knowledge, forward simulations have rarely been used to investigate the effect of stocking (but see Fernández‐Cebrián, Araguas, Sanz, & García‐Marín, 2014; Perrier, Guyomard et al., 2013). Here we assumed equal population size and used a small interval provided by LDNe estimates. However, estimates might be downwardly biased due to meta‐population structure, in which the true *N*_e_ is higher than in a single randomly mating population. Ideally, future studies will combine whole genome analysis with forward simulations integrating both positive and deleterious mutations, to better understand their effect on patterns of local ancestry (Kim, Huber, & Lohmueller, 2018).

Levels of population genetic diversity were modest, consistent with previous studies on Muskellunge based on microsatellite data in other geographic areas (Miller et al., 2017; Miller et al., 2012; Turnquist et al., 2017; Wilson et al., 2016). Although the various biases associated to RADseq data may complicate the interpretation of genetic diversity estimates (Arnold, Corbett‐Detig, Hartl, & Bomblies, 2013; Cariou, Duret, & Charlat, 2016; Gautier et al., 2013), some general patterns emerged from our data.

First, the population with the lowest level of genetic diversity (TRA) is strongly isolated, thus restricting opportunity for gene flow and implying small effective population size. Given the species life history traits, namely a long life span (15–30 years; Casselman, Robinson, & Crossman, 1999), a territorial and predatory behavior (Becker, 1983), small effective population sizes are expected (Romiguier et al., 2014).

Second, there was a significant and positive correlation between stocking ancestry and genetic diversity. Stocked populations from the Lower SLR and LDM, have been the most extensively stocked (>630,000 fish released) and displayed lower heterozygosity than remaining supplemented tributaries and inland lakes, or introduced populations from CHP, TRE, JOS, and FRO. Moreover, the most extensively stocked populations showed the lowest stocking ancestry. In Muskellunge, Scribner et al. (2015) found that stocked populations displayed higher genetic diversity when stocking was done using multiple strains. Similarly, Lake Trout (*Salvelinus namaycush*), Valiquette et al. (2014) and Ferchaud, Laporte, Perrier, and Bernatchez (2018) found that genetic diversity of stocked populations was higher than unstocked populations. Here, our results suggest that stocking could have some positive effects for populations found in the smaller tributaries and lakes. Some are currently disconnected from the St. Lawrence R. because of impassable dams. These populations are subject to drift due to their small effective population size that makes them prone to inbreeding depression (Sacherri et al., 1998; Wang et al., 1999). Stocking could contribute to reduce the probability of inbreeding depression by maintaining higher levels of genetic diversity (Frankham, 2015). Alternatively, it is unclear whether selection against introgression removed non‐native (e.g., stocked) alleles within the St. Lawrence or whether simple neutral admixture explains our results. For instance, Hansen et al. (2009) found that Danish Brown Trout (*Salmo trutta*) stocked for 60 years were subject to selection against non‐native alleles of stocking origin. Similarly, Muhlfeld et al. (2009) found a decline in fitness of Cutthroat Trout (*Oncorhynchus clarkii*) crossed with non‐native Rainbow Trout (*O. mykiss*) over generations. In our case, the time of divergence between stocked and population may not have been sufficient to generate differences in the load of deleterious mutations and to generate strong selection against introgression outcome. Therefore, it would be relevant for future studies to test whether selection against introgression was necessary and if it has been efficient in removal of non‐native alleles from stocked fish in the larger St. Lawrence as compared to smaller bottlenecked populations (Kim et al., 2018).

### 4.3 Conservation and management applications

Knowledge of the genetic structure of fish populations is essential for proper and sustainable stock management since it allows identification of groups of individuals that are genetically and often geographically unconnected, therefore implying at least some demographic independence. Our results indicate that Muskellunge samples studied here can be separated into the following groups: The first being the St. Lawrence watershed, characterized by an upstream‐to‐downstream pattern of dispersal and gene flow. The second group is des Deux‐Montagnes Lake with a shared co‐ancestry with the St. Lawrence R. population, especially fish from the north shore of Saint‐Louis L. The third group comprises tributaries draining into the St. Lawrence River. Each tributary has differentiated by genetic drift and display lower levels of polymorphism and lower effective population size possibly due to founder events. Therefore, ensuring the maintenance of connectivity of tributaries with the mainstem would contribute to maintenance of an overall large effective population size at the meta‐population scale, limiting the risks of inbreeding depression (Frankham, 2015). The fourth group is represented by lakes where Muskellunge has been introduced (Joseph L., Tremblant L. and Frontière L.). These populations exhibit high genetic similarity with the Chautauqua L. source of stocking. The fifth group includes stocked lakes where Muskellunge was initially present (Maskinongé and Champlain lakes) with each of these lakes representing a genetically distinct population. Finally, Traverse Lake represents the sixth group corresponding to a wild unstocked population with low genetic diversity. In isolated lacustrine and river systems originally unoccupied or with presumably low Muskellunge abundance, stocking efforts appear to have been successful in enhancing populations and contributed to support angling activity. This corroborates Dumont (1991) who reached similar conclusions following an analysis of data from the first half period of the stocking program in St. Louis L. located in the Lower St. Lawrence R.

In the smallest lakes, as well as in locations where a massive die‐off was observed (e.g., Thousand Islands; Farrell et al., 2017), supplementation may be necessary, and in such case, the most genetically diversified source should be favored.

In conclusion, our results show that Muskellunge populations are spatially structured into a set of different groups, whose differentiation was affected by the levels of stocking, natural connectivity, or geographic isolation. Our results highlight the necessity to go beyond the simple measurement of genetic differentiation and genetic structure to define subsequent management practice. We further suggest that fishery management and habitat protection measures should be applied globally in order to ensure high connectivity of the population and maintain their evolutionary potential at the highest. We also found that the genetic consequences of stocking on admixture and genetic diversity affected differentially Muskellunge from the St. Lawrence River, its tributaries, and inland lakes, whereby stocking apparently had little impact on the St. Lawrence R. and des Deux‐Montagnes L. populations but apparently led to more important admixture elsewhere. Disentangling the long‐term evolutionary consequences of this history of stocking would require further investigations from denser genome‐wide data.

## CONFLICT OF INTEREST

None declared.

## DATA AVAILABILITY

Raw sequence data are available at NCBI BioProject ID PRJNA512459. Pipeline for forward simulation is available on gitub at https://github.com/QuentinRougemont/fwd_sims.
