# Supplementary material for: Combining population genomics and forward simulations to investigate stocking impacts: A case study of Muskellunge (Esox masquinongy) from the St. Lawrence River basin
Source: Evol Appl. 2019 Jan 30;12(5):902–22. doi: 10.1111/eva.12765 (PMC6503833; doi:10.1111/eva.12765)
Supplement: Supplementary file 1 [file EVA-12-902-s001.pdf]

Supplementary Methods and results for

Combining population genomics and forward simulations to investigate stocking impacts: A case study of Muskellunge (*Esox masquinongy*) from the St. Lawrence River basin

Quentin Rougemont, Anne Carrier, Jeremy Leluyer, Anne-Laure Ferchaud, John M. Farrell, Daniel Hatin, Philippe Brodeur, Louis Bernatchez, 2019, Evolutionary Applications

Fig S1: A simplified representation of the stocking activity in the St. Lawrence water from 1950 until the end of stocking in 1997.
The varying intensity of the arrows depicts the intensity of stocking from the different source in each time periods. The dotted arrow from Pigeon lake reflects an unknown quantity of fishes introduced in the 1950-1965 time period.
The Table below extracted from De Lafontaine (unpublished results) displays the number of fish used for stocking in waters of the St Lawrence. Values in italic present detailed information for each sectors within the St.Lawrence River

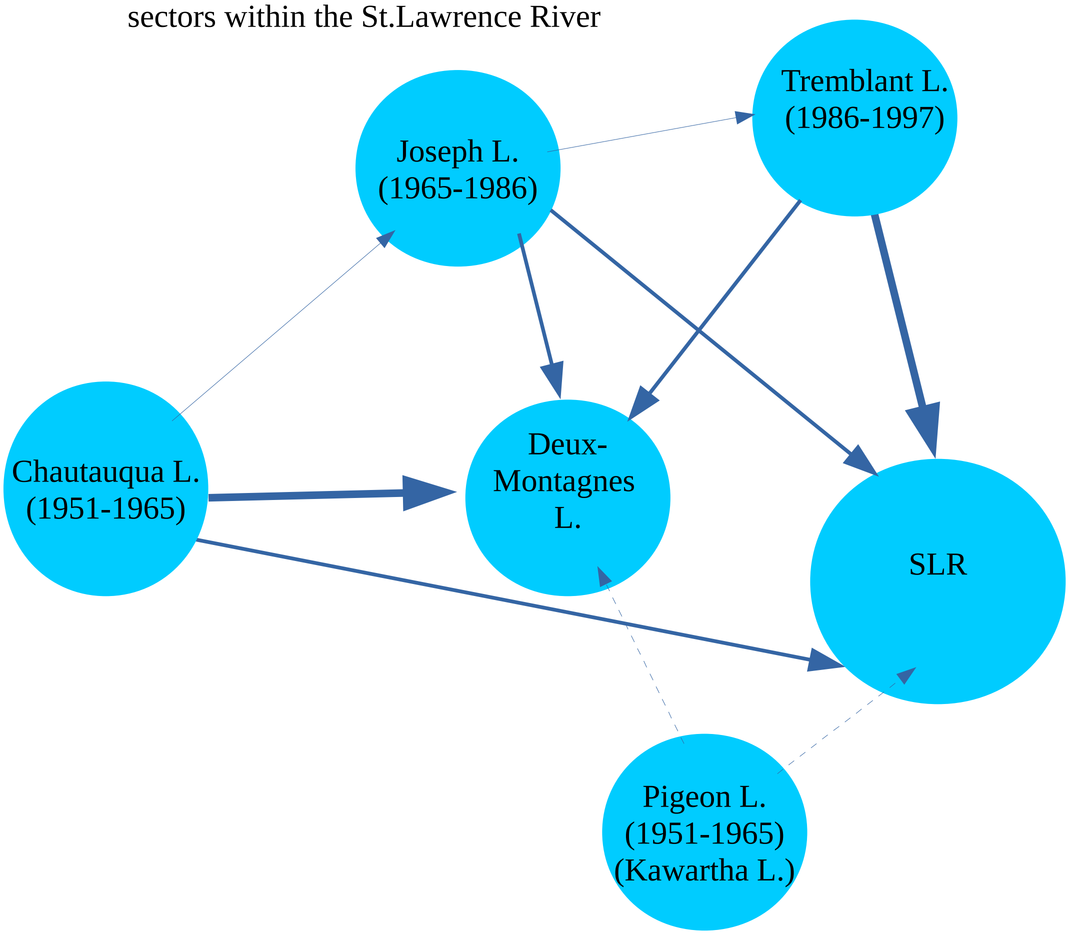

**Location**	**Larvae (< 5 cm)**	**Fry (5-27 cm)**	**Adults (> 27 cm)**	**Total**
**St.Lawrence River (SLR):**	**441,800**	**593,173**	**135**	**1,035,108**
Lake St.Francois		*77,494*	*2*	*77,49*
Beauharnois Canal & Soulanges Canal	*30,000*	*11,250*		*41,250*
Lake St.Louis	*271,800*	*168,980*	*133*	*440,913*
Lake Deux-Montagnes	*20,000*	*81,692*		*101,692*
Des Mille-Isles / Des Prairies Rivers		*38,297*		*38,297*
River stretch (downstream Montreal)	*120,000*	*208,160*		*328,160*
Lake St.Pierre		*7,300*		*7,300*
Inland lakes & rivers – North of SLR	300,000	74,880		329,880
Inland lakes & rivers – South of SLR		17,825	2	17,827
Tributaries of SLR	112,000	88,908	60	200,968
**All**	**853,800**	**729,786**	**197**	**1,583,108**

Fig S2: Isolation by Distance within the St. Lawrence

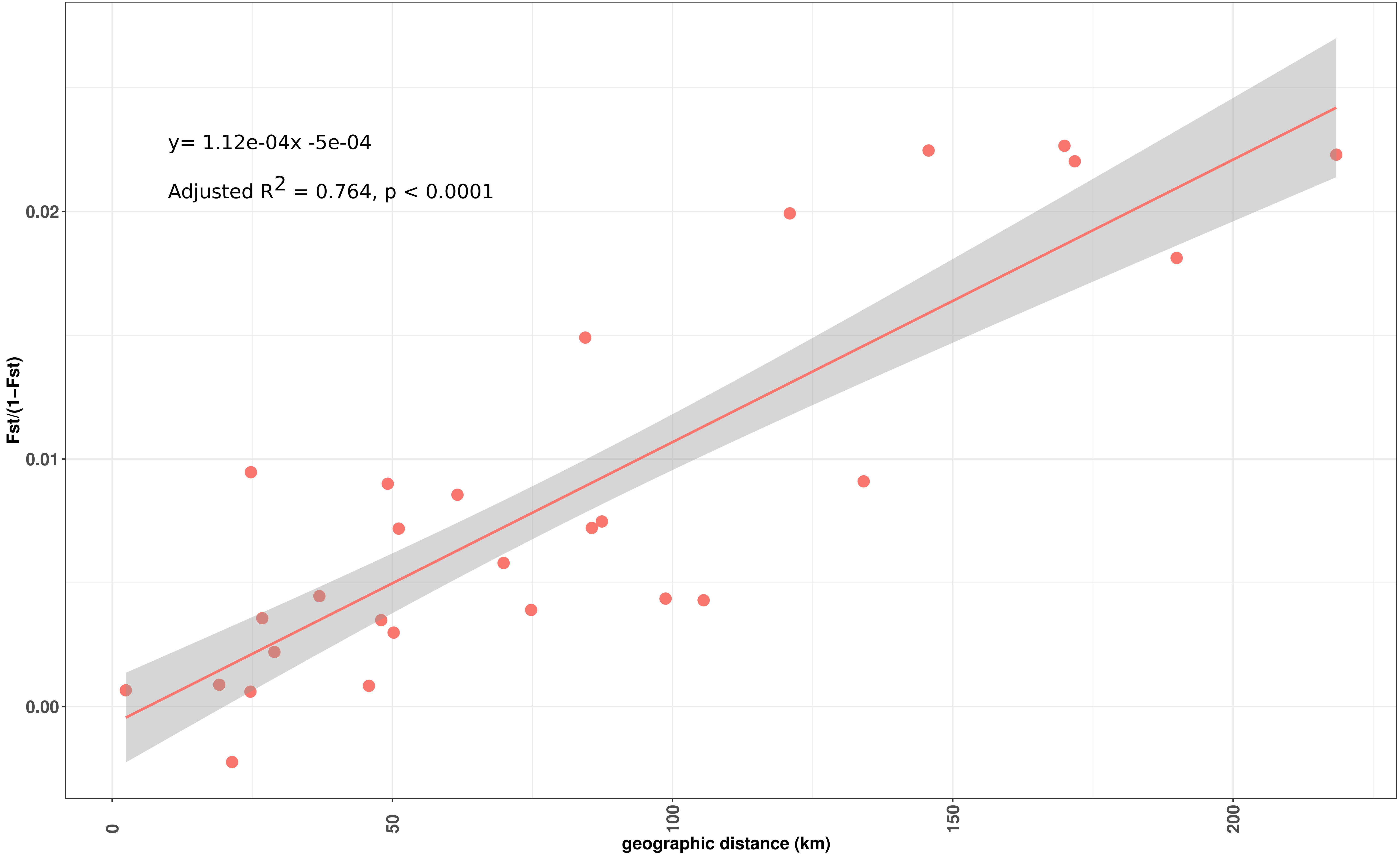

Figure S3: (a) admixture Cross-validation plot obtained for the 643 individuals and different K values.
(b) admixture plots for different K values.

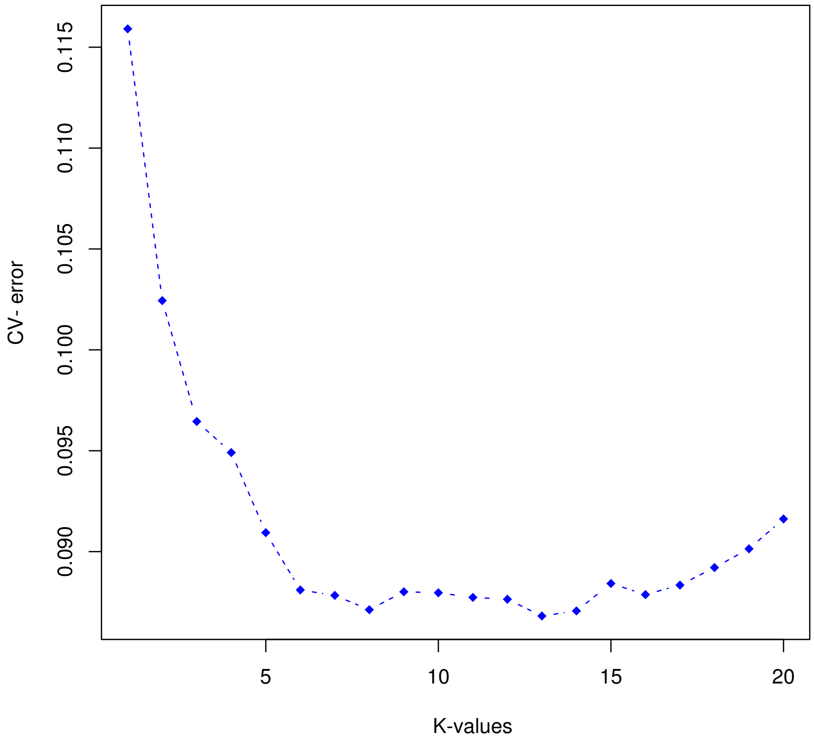

a)

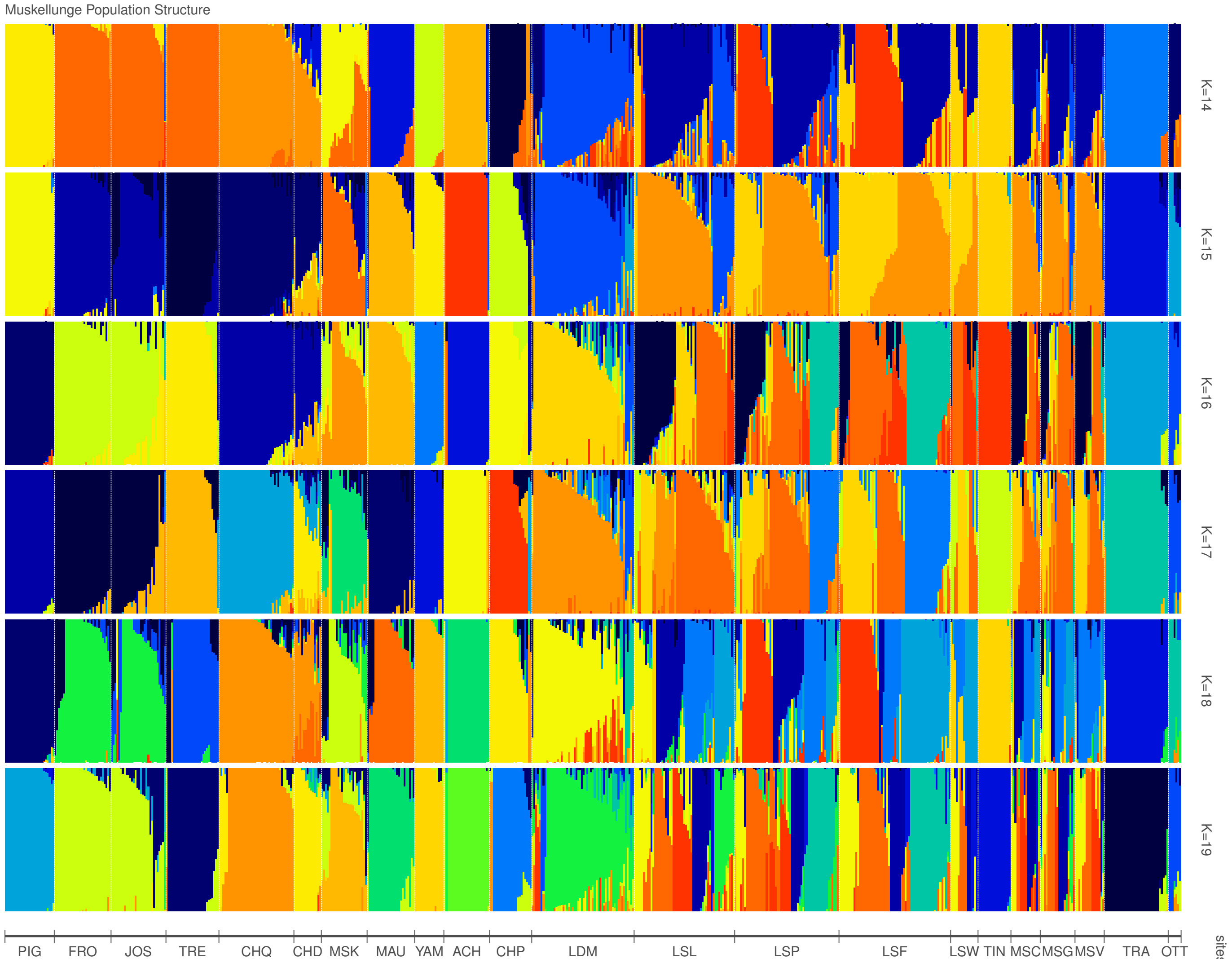

b)

Figure S4: Simulated models of divergence with admixture.

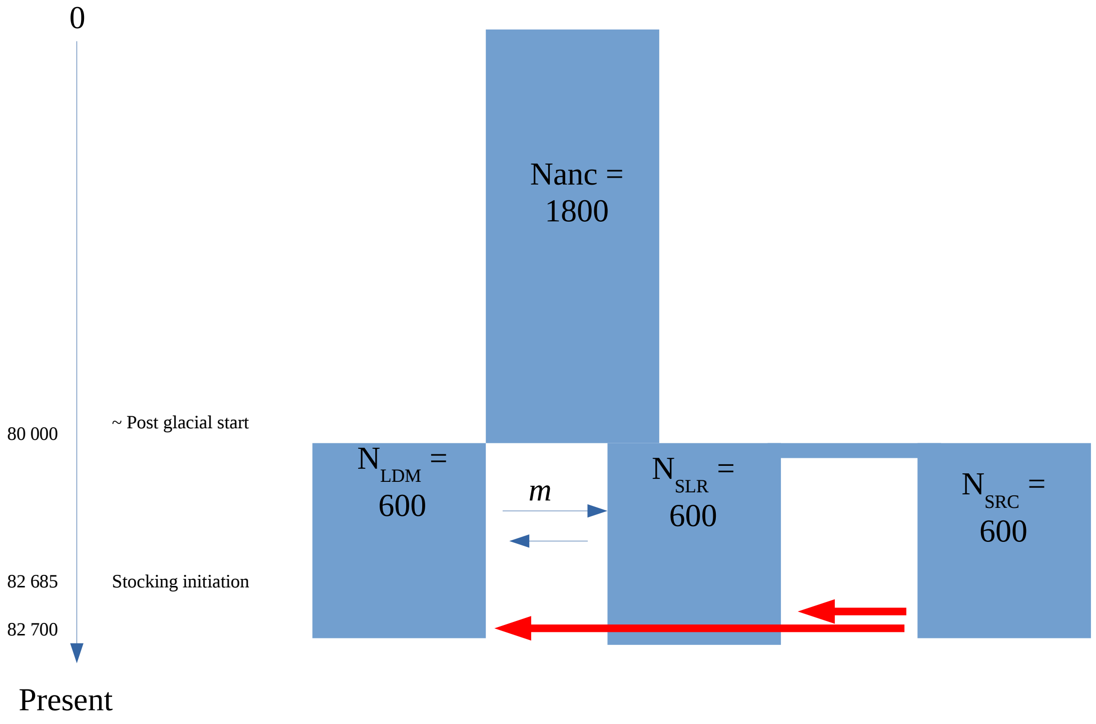

	popA	popB	sxA_avg	sxB_avg	ss_avg	piA_avg	piB_avg	divAB_avg	netdivAB_avg	FST_avg
1	CHD	ACH	0.205	0.286	0.751	0.0032014	0.0030203	0.0038205	0.0007097	0.0385870
2	CHP	ACH	0.198	0.284	0.774	0.0031345	0.0030831	0.0037389	0.0006301	0.0593632
3	CHP	CHD	0.215	0.216	0.814	0.0033180	0.0034589	0.0038798	0.0004914	0.0144615
4	CHQ	ACH	0.283	0.242	0.747	0.0028121	0.0028858	0.0036830	0.0008341	0.0563876
5	CHQ	CHD	0.278	0.152	0.838	0.0030421	0.0033182	0.0032695	0.0000893	−0.0733390
6	CHQ	CHP	0.289	0.168	0.828	0.0030493	0.0031962	0.0036785	0.0005558	0.0507369
7	FRO	ACH	0.236	0.298	0.736	0.0028773	0.0030074	0.0038473	0.0009049	0.0758679
8	FRO	CHD	0.221	0.197	0.843	0.0031494	0.0034913	0.0034753	0.0001550	−0.0492876
9	FRO	CHP	0.229	0.201	0.853	0.0031961	0.0033988	0.0038944	0.0005970	0.0557195
10	FRO	CHQ	0.148	0.252	0.895	0.0030812	0.0031284	0.0032449	0.0001401	0.0180456
11	JOS	ACH	0.254	0.234	0.773	0.0028505	0.0029349	0.0037352	0.0008425	0.0739751
12	JOS	CHD	0.263	0.163	0.828	0.0030355	0.0033192	0.0033171	0.0001398	−0.0256134
13	JOS	CHP	0.272	0.171	0.828	0.0030589	0.0032047	0.0036815	0.0005497	0.0564989
14	JOS	CHQ	0.181	0.203	0.892	0.0029813	0.0029800	0.0030750	0.0000944	0.0015817
15	JOS	FRO	0.228	0.140	0.909	0.0031623	0.0031127	0.0031743	0.0000368	0.0027899
16	LDM	ACH	0.377	0.082	0.820	0.0030772	0.0026210	0.0031683	0.0003192	0.0715963
17	LDM	CHD	0.438	0.080	0.756	0.0030769	0.0027878	0.0032734	0.0003410	0.0104840
18	LDM	CHP	0.449	0.084	0.749	0.0030705	0.0026623	0.0032138	0.0003474	0.0686423
19	LDM	CHQ	0.363	0.116	0.805	0.0030115	0.0024964	0.0031726	0.0004187	0.0619425
20	LDM	FRO	0.449	0.111	0.729	0.0030352	0.0024765	0.0032421	0.0004862	0.0780516
21	LDM	JOS	0.362	0.092	0.825	0.0030576	0.0025319	0.0032187	0.0004240	0.0739248
22	LSF	ACH	0.369	0.122	0.801	0.0030488	0.0026764	0.0032729	0.0004103	0.0502516
23	LSF	CHD	0.453	0.144	0.683	0.0029540	0.0027575	0.0033641	0.0005084	−0.0337684
24	LSF	CHP	0.447	0.139	0.697	0.0029891	0.0026809	0.0032986	0.0004637	0.0435096
25	LSF	CHQ	0.375	0.175	0.738	0.0028943	0.0024718	0.0032870	0.0006039	0.0752434
26	LSF	FRO	0.457	0.167	0.668	0.0029293	0.0024592	0.0033726	0.0006784	0.0804195
27	LSF	JOS	0.385	0.164	0.737	0.0029221	0.0024924	0.0033140	0.0006067	0.0716988
28	LSF	LDM	0.162	0.208	0.928	0.0028330	0.0029214	0.0029724	0.0000952	0.0094015
29	LSL	ACH	0.384	0.075	0.820	0.0030694	0.0025979	0.0031718	0.0003381	0.0787662
30	LSL	CHD	0.447	0.078	0.744	0.0030409	0.0027388	0.0032828	0.0003929	0.0235034
31	LSL	CHP	0.457	0.080	0.747	0.0030573	0.0026379	0.0032174	0.0003698	0.0767192
32	LSL	CHQ	0.397	0.137	0.756	0.0029403	0.0024210	0.0031847	0.0005041	0.0704910
33	LSL	FRO	0.454	0.101	0.726	0.0030126	0.0024393	0.0032709	0.0005450	0.0875128
34	LSL	JOS	0.397	0.114	0.777	0.0029804	0.0024570	0.0032254	0.0005067	0.0792839
35	LSL	LDM	0.172	0.152	0.976	0.0029138	0.0028941	0.0029635	0.0000596	0.0066376
36	LSL	LSF	0.212	0.137	0.949	0.0029542	0.0028438	0.0029270	0.0000280	−0.0007533
37	LSP	ACH	0.387	0.092	0.807	0.0030128	0.0026120	0.0031770	0.0003646	0.0686410
38	LSP	CHD	0.458	0.101	0.711	0.0029531	0.0027183	0.0032811	0.0004454	−0.0033252
39	LSP	CHP	0.452	0.094	0.733	0.0029961	0.0026453	0.0032412	0.0004206	0.0616424
40	LSP	CHQ	0.376	0.134	0.775	0.0029041	0.0024550	0.0032181	0.0005386	0.0712683
41	LSP	FRO	0.467	0.126	0.700	0.0029269	0.0024295	0.0032812	0.0006030	0.0831387
42	LSP	JOS	0.380	0.114	0.786	0.0029455	0.0024838	0.0032536	0.0005390	0.0764743
43	LSP	LDM	0.160	0.155	0.979	0.0028656	0.0029154	0.0029548	0.0000644	0.0076327
44	LSP	LSF	0.178	0.122	0.994	0.0029538	0.0029146	0.0029424	0.0000082	−0.0005377
45	LSP	LSL	0.147	0.160	0.997	0.0028731	0.0029423	0.0029257	0.0000180	0.0015614
46	MAS	ACH	0.232	0.227	0.794	0.0035021	0.0029801	0.0036938	0.0004527	−0.0037864
47	MAS	CHD	0.311	0.235	0.686	0.0034193	0.0030953	0.0038195	0.0005622	0.0540048
48	MAS	CHP	0.316	0.237	0.694	0.0034543	0.0029962	0.0037492	0.0005240	0.0105888
49	MAS	CHQ	0.278	0.308	0.677	0.0032554	0.0026907	0.0036574	0.0006843	−0.0910666
50	MAS	FRO	0.334	0.274	0.651	0.0033667	0.0027397	0.0037931	0.0007399	−0.0279082
51	MAS	JOS	0.274	0.284	0.697	0.0033083	0.0027291	0.0037068	0.0006881	−0.0188995
52	MAS	LDM	0.085	0.372	0.818	0.0030758	0.0030679	0.0031963	0.0001245	−0.0304527
53	MAS	LSF	0.056	0.308	0.913	0.0032992	0.0031972	0.0032664	0.0000182	−0.0275856
54	MAS	LSL	0.048	0.362	0.855	0.0030840	0.0030989	0.0031077	0.0000163	0.0165756
55	MAS	LSP	0.060	0.355	0.861	0.0031338	0.0030722	0.0031346	0.0000316	−0.0050888
56	MAU	ACH	0.228	0.292	0.746	0.0030601	0.0030268	0.0038421	0.0007986	0.0733964
57	MAU	CHD	0.194	0.183	0.875	0.0033795	0.0035532	0.0036554	0.0001890	−0.0153591
58	MAU	CHP	0.236	0.220	0.816	0.0033105	0.0033370	0.0039019	0.0005782	0.0555716
59	MAU	CHQ	0.174	0.285	0.827	0.0031487	0.0030270	0.0033542	0.0002663	0.0229764
60	MAU	FRO	0.185	0.201	0.895	0.0034114	0.0032352	0.0035591	0.0002358	0.0239821
61	MAU	JOS	0.160	0.263	0.853	0.0031995	0.0030814	0.0033488	0.0002084	0.0257194
62	MAU	LDM	0.104	0.443	0.724	0.0026143	0.0030370	0.0032493	0.0004237	0.0693781
63	MAU	LSF	0.157	0.457	0.680	0.0026261	0.0029601	0.0033957	0.0006027	0.0637479
64	MAU	LSL	0.094	0.452	0.734	0.0025900	0.0030238	0.0032872	0.0004803	0.0797389
65	MAU	LSP	0.118	0.460	0.707	0.0025843	0.0029466	0.0032982	0.0005327	0.0708431
66	MAU	MAS	0.262	0.327	0.666	0.0029212	0.0033993	0.0038307	0.0006704	0.0038742
67	MIL	ACH	0.235	0.226	0.798	0.0033860	0.0029771	0.0036553	0.0004738	0.0223787
68	MIL	CHD	0.308	0.224	0.711	0.0033429	0.0031212	0.0037963	0.0005642	0.0573003
69	MIL	CHP	0.311	0.227	0.714	0.0033671	0.0030151	0.0037214	0.0005303	0.0343833
70	MIL	CHQ	0.271	0.299	0.696	0.0031699	0.0027080	0.0036310	0.0006920	−0.0353158
71	MIL	FRO	0.324	0.255	0.682	0.0032975	0.0027758	0.0037942	0.0007576	0.0133461
72	MIL	JOS	0.264	0.274	0.717	0.0032242	0.0027580	0.0036952	0.0007041	0.0197237
73	MIL	LDM	0.089	0.376	0.820	0.0029753	0.0030681	0.0031701	0.0001484	−0.0013795
74	MIL	LSF	0.077	0.319	0.884	0.0031487	0.0031483	0.0032093	0.0000608	−0.0144435
75	MIL	LSL	0.056	0.364	0.855	0.0029799	0.0030954	0.0030894	0.0000518	0.0293529
76	MIL	LSP	0.071	0.363	0.847	0.0030091	0.0030502	0.0030962	0.0000665	0.0117672
77	MIL	MAS	0.180	0.173	0.895	0.0035302	0.0036474	0.0036231	0.0000343	0.0013384
78	MIL	MAU	0.318	0.248	0.693	0.0033213	0.0029556	0.0038145	0.0006760	0.0339241
79	MSC	ACH	0.246	0.201	0.796	0.0034033	0.0029079	0.0035435	0.0003879	−0.0083523
80	MSC	CHD	0.314	0.201	0.716	0.0033676	0.0030650	0.0037014	0.0004851	0.0497782
81	MSC	CHP	0.327	0.203	0.716	0.0034022	0.0029579	0.0036239	0.0004439	0.0068896
82	MSC	CHQ	0.281	0.267	0.716	0.0032300	0.0026753	0.0035577	0.0006050	−0.0869112
83	MSC	FRO	0.339	0.236	0.682	0.0033245	0.0027180	0.0036754	0.0006542	−0.0271725
84	MSC	JOS	0.267	0.244	0.739	0.0032845	0.0027287	0.0036172	0.0006106	−0.0174102
85	MSC	LDM	0.083	0.330	0.862	0.0030710	0.0030775	0.0031555	0.0000812	−0.0309826
86	MSC	LSF	0.093	0.293	0.888	0.0031856	0.0030955	0.0031639	0.0000233	−0.0677176
87	MSC	LSL	0.053	0.323	0.896	0.0030788	0.0031073	0.0030926	−0.0000004	0.0054298
88	MSC	LSP	0.065	0.319	0.892	0.0031087	0.0030626	0.0031013	0.0000157	−0.0174391
89	MSC	MAS	0.200	0.157	0.882	0.0035345	0.0035514	0.0035510	0.0000080	0.0026766
90	MSC	MAU	0.327	0.223	0.703	0.0033619	0.0029054	0.0037190	0.0005853	0.0019818
91	MSC	MIL	0.198	0.163	0.882	0.0035193	0.0034199	0.0035095	0.0000399	0.0013146
92	MSG	ACH	0.267	0.163	0.822	0.0033884	0.0028737	0.0034985	0.0003674	0.0086798
93	MSG	CHD	0.338	0.160	0.741	0.0033551	0.0030209	0.0036101	0.0004221	0.0507400
94	MSG	CHP	0.343	0.169	0.744	0.0033843	0.0029252	0.0035695	0.0004148	0.0232618
95	MSG	CHQ	0.295	0.229	0.741	0.0032092	0.0026436	0.0034625	0.0005361	−0.0548301
96	MSG	FRO	0.360	0.204	0.703	0.0032986	0.0026767	0.0035659	0.0005782	−0.0032537
97	MSG	JOS	0.289	0.202	0.768	0.0032791	0.0027010	0.0035308	0.0005407	0.0022561
98	MSG	LDM	0.094	0.293	0.895	0.0030696	0.0030566	0.0031312	0.0000682	−0.0232905
99	MSG	LSF	0.119	0.271	0.892	0.0031368	0.0030300	0.0031093	0.0000258	−0.0642031
100	MSG	LSL	0.069	0.287	0.918	0.0030649	0.0030712	0.0030654	−0.0000027	−0.0011883
101	MSG	LSP	0.083	0.281	0.915	0.0030988	0.0030335	0.0030819	0.0000158	−0.0207967
102	MSG	MAS	0.242	0.134	0.868	0.0034541	0.0034359	0.0034556	0.0000105	0.0066570
103	MSG	MAU	0.351	0.179	0.723	0.0033358	0.0028537	0.0036159	0.0005212	0.0185452
104	MSG	MIL	0.247	0.147	0.858	0.0034321	0.0033060	0.0034205	0.0000514	0.0081986
105	MSG	MSC	0.197	0.143	0.899	0.0034087	0.0033895	0.0033921	−0.0000071	0.0010681
106	MSK	ACH	0.270	0.225	0.781	0.0032230	0.0029289	0.0036098	0.0005339	0.0518805
107	MSK	CHD	0.271	0.147	0.842	0.0034207	0.0033088	0.0036196	0.0002549	0.0163822
108	MSK	CHP	0.291	0.158	0.828	0.0034252	0.0031677	0.0037412	0.0004448	0.0458362
109	MSK	CHQ	0.230	0.217	0.844	0.0032773	0.0028949	0.0034177	0.0003316	0.0028973
110	MSK	FRO	0.256	0.144	0.883	0.0034942	0.0030407	0.0035859	0.0003185	0.0193919
111	MSK	JOS	0.219	0.190	0.871	0.0033301	0.0029429	0.0034244	0.0002879	0.0230240
112	MSK	LDM	0.128	0.370	0.793	0.0028062	0.0029922	0.0031498	0.0002507	0.0240020
113	MSK	LSF	0.187	0.377	0.728	0.0027816	0.0028727	0.0032017	0.0003746	0.0016484
114	MSK	LSL	0.125	0.383	0.785	0.0027660	0.0029676	0.0031517	0.0002850	0.0342210
115	MSK	LSP	0.140	0.386	0.767	0.0027635	0.0028953	0.0031534	0.0003240	0.0195307
116	MSK	MAS	0.291	0.254	0.714	0.0030847	0.0032994	0.0035921	0.0004000	0.0115440
117	MSK	MAU	0.275	0.153	0.857	0.0034626	0.0031809	0.0036626	0.0003408	0.0324395
118	MSK	MIL	0.286	0.248	0.732	0.0031073	0.0032130	0.0035805	0.0004204	0.0328650
119	MSK	MSC	0.257	0.259	0.739	0.0030512	0.0032478	0.0034873	0.0003378	0.0084812
120	MSK	MSG	0.216	0.277	0.774	0.0030343	0.0032546	0.0034346	0.0002901	0.0170921
121	MSV	ACH	0.252	0.183	0.816	0.0035035	0.0029160	0.0036237	0.0004139	−0.0098118
122	MSV	CHD	0.324	0.179	0.730	0.0034622	0.0030534	0.0037307	0.0004729	0.0494927
123	MSV	CHP	0.333	0.183	0.733	0.0034933	0.0029529	0.0036728	0.0004497	0.0045771
124	MSV	CHQ	0.284	0.264	0.718	0.0032888	0.0026644	0.0035678	0.0005911	−0.0927010
125	MSV	FRO	0.343	0.222	0.700	0.0034098	0.0027146	0.0037001	0.0006378	−0.0325348
126	MSV	JOS	0.284	0.238	0.740	0.0033522	0.0027074	0.0036251	0.0005953	−0.0230919
127	MSV	LDM	0.093	0.325	0.867	0.0031365	0.0030600	0.0031944	0.0000962	−0.0453730
128	MSV	LSF	0.103	0.280	0.899	0.0032748	0.0030913	0.0032053	0.0000222	−0.0800394
129	MSV	LSL	0.058	0.309	0.908	0.0031529	0.0030985	0.0031280	0.0000023	−0.0053232
130	MSV	LSP	0.077	0.307	0.894	0.0031722	0.0030419	0.0031260	0.0000189	−0.0335352
131	MSV	MAS	0.210	0.141	0.893	0.0036146	0.0035348	0.0035796	0.0000049	0.0029493
132	MSV	MAU	0.339	0.205	0.713	0.0034447	0.0028926	0.0037465	0.0005778	−0.0015552
133	MSV	MIL	0.215	0.147	0.881	0.0035920	0.0033950	0.0035430	0.0000494	0.0011880
134	MSV	MSC	0.177	0.153	0.908	0.0035596	0.0034678	0.0035087	−0.0000050	0.0004545
135	MSV	MSG	0.159	0.191	0.895	0.0034595	0.0033874	0.0034201	−0.0000033	−0.0001268
136	MSV	MSK	0.267	0.249	0.748	0.0033276	0.0030469	0.0035344	0.0003472	0.0071730
137	ONT	ACH	0.181	0.501	0.585	0.0019118	0.0031683	0.0039874	0.0014474	0.1158014
138	ONT	CHD	0.201	0.456	0.595	0.0020056	0.0035256	0.0041565	0.0013909	0.0160322
139	ONT	CHP	0.220	0.472	0.572	0.0019874	0.0033549	0.0042612	0.0015901	0.1188597
140	ONT	CHQ	0.176	0.531	0.572	0.0018658	0.0030039	0.0040302	0.0015954	0.1648240
141	ONT	FRO	0.188	0.468	0.618	0.0020241	0.0032131	0.0043537	0.0017351	0.1594679
142	ONT	JOS	0.173	0.511	0.588	0.0019077	0.0030706	0.0040661	0.0015769	0.1451907
143	ONT	LDM	0.104	0.660	0.528	0.0015679	0.0030475	0.0032845	0.0009768	0.1722403
144	ONT	LSF	0.110	0.635	0.548	0.0016331	0.0030775	0.0034443	0.0010889	0.1755705
145	ONT	LSL	0.089	0.658	0.536	0.0015543	0.0030380	0.0032619	0.0009658	0.1817642
146	ONT	LSP	0.105	0.655	0.529	0.0015638	0.0029811	0.0032710	0.0009986	0.1758420
147	ONT	MAS	0.185	0.510	0.552	0.0018565	0.0036347	0.0039710	0.0012255	−0.0235603
148	ONT	MAU	0.195	0.459	0.621	0.0020403	0.0034090	0.0042767	0.0015521	0.1319314
149	ONT	MIL	0.175	0.506	0.585	0.0018975	0.0035714	0.0039542	0.0012197	0.0293597
150	ONT	MSC	0.173	0.532	0.541	0.0017992	0.0034972	0.0037861	0.0011379	−0.0187260
151	ONT	MSG	0.156	0.556	0.543	0.0017470	0.0034102	0.0036747	0.0010961	0.0263817
152	ONT	MSK	0.159	0.520	0.606	0.0018945	0.0034497	0.0039241	0.0012520	0.1038830
153	ONT	MSV	0.166	0.539	0.556	0.0018019	0.0035813	0.0038445	0.0011529	−0.0210538
154	TRA	ACH	0.169	0.409	0.690	0.0016868	0.0031948	0.0038071	0.0013664	0.0615567
155	TRA	CHD	0.216	0.381	0.659	0.0017255	0.0034733	0.0042162	0.0016168	−0.0724945
156	TRA	CHP	0.218	0.369	0.686	0.0017696	0.0034118	0.0041634	0.0015728	0.0650849
157	TRA	CHQ	0.152	0.436	0.688	0.0016582	0.0030556	0.0040456	0.0016887	0.1628309
158	TRA	FRO	0.212	0.399	0.678	0.0017563	0.0031856	0.0043245	0.0018535	0.1400106
159	TRA	JOS	0.159	0.420	0.692	0.0016729	0.0030865	0.0040783	0.0016986	0.1178408
160	TRA	LDM	0.066	0.564	0.653	0.0014059	0.0031252	0.0032673	0.0010017	0.1734010
161	TRA	LSF	0.090	0.549	0.642	0.0014402	0.0031112	0.0033746	0.0010989	0.1766408
162	TRA	LSL	0.079	0.585	0.623	0.0013731	0.0030728	0.0032412	0.0010182	0.1709042
163	TRA	LSP	0.069	0.559	0.648	0.0014034	0.0030585	0.0032545	0.0010236	0.1736674
164	TRA	MAS	0.198	0.433	0.619	0.0016088	0.0035912	0.0038682	0.0012682	−0.1216717
165	TRA	MAU	0.212	0.386	0.684	0.0017564	0.0033601	0.0042796	0.0017214	0.1014230
166	TRA	MIL	0.186	0.429	0.641	0.0016265	0.0035138	0.0038766	0.0013065	−0.0447828
167	TRA	MSC	0.171	0.447	0.637	0.0015862	0.0035273	0.0037356	0.0011789	−0.1192391
168	TRA	MSG	0.151	0.477	0.632	0.0015394	0.0034504	0.0036216	0.0011267	−0.0639497
169	TRA	MSK	0.156	0.440	0.683	0.0016482	0.0034390	0.0037571	0.0012134	0.0406647
170	TRA	MSV	0.165	0.466	0.632	0.0015729	0.0035921	0.0037725	0.0011899	−0.1241551
171	TRA	ONT	0.392	0.291	0.571	0.0018908	0.0021664	0.0045289	0.0025003	0.1912753
172	TRE	ACH	0.217	0.333	0.711	0.0028569	0.0030491	0.0039097	0.0009567	0.0806607
173	TRE	CHD	0.202	0.248	0.812	0.0031242	0.0035497	0.0035055	0.0001685	−0.0552367
174	TRE	CHP	0.195	0.238	0.834	0.0032015	0.0034841	0.0039974	0.0006546	0.0570675
175	TRE	CHQ	0.125	0.299	0.869	0.0030653	0.0031867	0.0032747	0.0001487	0.0280690
176	TRE	FRO	0.127	0.202	0.967	0.0033726	0.0034563	0.0035186	0.0001042	0.0128397
177	TRE	JOS	0.130	0.284	0.866	0.0030718	0.0031959	0.0032112	0.0000773	0.0108797
178	TRE	LDM	0.100	0.491	0.693	0.0024329	0.0030549	0.0032594	0.0005155	0.0900744
179	TRE	LSF	0.157	0.496	0.639	0.0024243	0.0029527	0.0034000	0.0007115	0.0907540
180	TRE	LSL	0.088	0.503	0.693	0.0024003	0.0030372	0.0032981	0.0005794	0.1003403
181	TRE	LSP	0.116	0.503	0.666	0.0023983	0.0029579	0.0033167	0.0006386	0.0939044
182	TRE	MAS	0.252	0.369	0.631	0.0027134	0.0034197	0.0038492	0.0007826	−0.0209169
183	TRE	MAU	0.175	0.238	0.864	0.0032136	0.0034786	0.0036205	0.0002744	0.0267870
184	TRE	MIL	0.249	0.368	0.646	0.0027490	0.0033380	0.0038512	0.0008077	0.0213735
185	TRE	MSC	0.221	0.380	0.651	0.0026959	0.0033719	0.0037333	0.0006994	−0.0206856
186	TRE	MSG	0.183	0.395	0.675	0.0026498	0.0033401	0.0036175	0.0006225	0.0052278
187	TRE	MSK	0.135	0.312	0.838	0.0029917	0.0035230	0.0036306	0.0003732	0.0259004
188	TRE	MSV	0.205	0.387	0.661	0.0026797	0.0034510	0.0037497	0.0006844	−0.0253675
189	TRE	ONT	0.447	0.232	0.585	0.0031774	0.0020511	0.0044691	0.0018548	0.1618313
190	TRE	TRA	0.372	0.252	0.660	0.0031857	0.0017992	0.0044388	0.0019464	0.1402268
191	YAM	ACH	0.169	0.317	0.770	0.0031138	0.0031670	0.0037747	0.0006344	0.0541483
192	YAM	CHD	0.206	0.274	0.754	0.0031968	0.0034555	0.0041369	0.0008107	0.0695933
193	YAM	CHP	0.199	0.278	0.781	0.0032602	0.0033879	0.0040942	0.0007701	0.0652183
194	YAM	CHQ	0.173	0.356	0.741	0.0030286	0.0029935	0.0039307	0.0009196	0.0464905
195	YAM	FRO	0.216	0.301	0.755	0.0032153	0.0031305	0.0041586	0.0009857	0.0701960
196	YAM	JOS	0.176	0.339	0.749	0.0030568	0.0030176	0.0039519	0.0009147	0.0737255
197	YAM	LDM	0.068	0.492	0.717	0.0025963	0.0031043	0.0032756	0.0004254	0.0681979
198	YAM	LSF	0.113	0.487	0.681	0.0026269	0.0030429	0.0033505	0.0005156	0.0202252
199	YAM	LSL	0.058	0.495	0.718	0.0025719	0.0030932	0.0032635	0.0004310	0.0821366
200	YAM	LSP	0.082	0.499	0.697	0.0025747	0.0030175	0.0032706	0.0004744	0.0546913
201	YAM	MAS	0.198	0.345	0.696	0.0029737	0.0035553	0.0038380	0.0005735	0.0498371
202	YAM	MAU	0.211	0.298	0.753	0.0032022	0.0032963	0.0041680	0.0009187	0.0748995
203	YAM	MIL	0.182	0.339	0.721	0.0029962	0.0034726	0.0038374	0.0006030	0.0628365
204	YAM	MSC	0.157	0.351	0.722	0.0029295	0.0035012	0.0037346	0.0005192	0.0489057
205	YAM	MSG	0.142	0.378	0.720	0.0028534	0.0034205	0.0036151	0.0004781	0.0590800
206	YAM	MSK	0.156	0.345	0.762	0.0030454	0.0034091	0.0038771	0.0006498	0.0746721
207	YAM	MSV	0.151	0.362	0.724	0.0029080	0.0035661	0.0037564	0.0005193	0.0497268
208	YAM	ONT	0.432	0.246	0.570	0.0033191	0.0020428	0.0043578	0.0016768	0.0640918
209	YAM	TRA	0.348	0.250	0.665	0.0033562	0.0018015	0.0042624	0.0016835	−0.0129744
210	YAM	TRE	0.254	0.275	0.736	0.0032888	0.0031284	0.0042786	0.0010700	0.0703449
211	OUT	ACH	0.144	0.388	0.705	0.0035745	0.0032027	0.0039546	0.0005660	−0.0623304
212	OUT	CHD	0.150	0.332	0.746	0.0037700	0.0036125	0.0040659	0.0003746	0.0195642
213	OUT	CHP	0.174	0.347	0.728	0.0037797	0.0034643	0.0041676	0.0005456	−0.0573576
214	OUT	CHQ	0.135	0.425	0.702	0.0035183	0.0030768	0.0037852	0.0004877	−0.1765928
215	OUT	FRO	0.182	0.371	0.702	0.0037193	0.0032057	0.0040007	0.0005383	−0.1314545
216	OUT	JOS	0.132	0.402	0.712	0.0035460	0.0031093	0.0038132	0.0004856	−0.0918631
217	OUT	LDM	0.038	0.557	0.674	0.0029944	0.0031705	0.0033043	0.0002218	−0.0163542
218	OUT	LSF	0.085	0.559	0.632	0.0030140	0.0030957	0.0034125	0.0003576	−0.1647621
219	OUT	LSL	0.039	0.569	0.655	0.0029218	0.0031187	0.0032864	0.0002662	−0.0132900
220	OUT	LSP	0.052	0.557	0.649	0.0029529	0.0030630	0.0033211	0.0003132	−0.0748890
221	OUT	MAS	0.153	0.401	0.671	0.0034641	0.0036618	0.0039643	0.0004013	0.0355293
222	OUT	MAU	0.173	0.363	0.724	0.0037742	0.0034298	0.0040634	0.0004614	−0.0842553
223	OUT	MIL	0.149	0.401	0.677	0.0034781	0.0035582	0.0039182	0.0004000	0.0170616
224	OUT	MSC	0.124	0.413	0.681	0.0033930	0.0035745	0.0038251	0.0003413	0.0392643
225	OUT	MSG	0.099	0.437	0.697	0.0033497	0.0035439	0.0037418	0.0002950	0.0349841
226	OUT	MSK	0.112	0.415	0.728	0.0035323	0.0035112	0.0038394	0.0003176	−0.0187652
227	OUT	MSV	0.118	0.431	0.682	0.0033609	0.0036519	0.0038425	0.0003361	0.0444350
228	OUT	ONT	0.399	0.312	0.535	0.0039337	0.0021328	0.0042946	0.0012613	−0.2859461
229	TRE	OUT	0.354	0.224	0.680	0.0031989	0.0038056	0.0041124	0.0006101	−0.1398444
230	TRA	OUT	0.318	0.307	0.626	0.0018586	0.0039192	0.0044268	0.0015378	−0.4189416
231	YAM	OUT	0.319	0.210	0.701	0.0033912	0.0038403	0.0043382	0.0007224	−0.0035782

Table S2. summary statistics for the 80pb haplotype data obtained from stacks.

sx= number of polymorphic sites private to each population
ss= number of shared polymorphic sites
pi= nucleotide diversity (Tajima, 1989)
divAB = Dxy
netDiv = divergence net
Fst= between population differentiation

Table S1: pairwise Weir & CockerhamFst values.

Non significant Fst values are in bold

	ACH	CHD	CHP	CHQ	FRO	JOS	LDM	LSF	LSL	LSP	LSW	MAU	TIN	MSC	MSG	MSK	MSV	PIG	OTT	TRA	TRE
**CHD**	0.250																				
**CHP**	0.222	0.172																			
**CHQ**	0.290	0.039	0.198																		
**FRO**	0.309	0.073	0.207	0.056																	
**JOS**	0.291	0.063	0.195	0.047	0.013																
**LDM**	0.124	0.129	0.135	0.168	0.185	0.166															
**LSF**	0.156	0.193	0.178	0.235	0.252	0.233	0.044														
**LSL**	0.136	0.158	0.149	0.204	0.216	0.200	0.023	0.009													
**LSP**	0.144	0.172	0.162	0.216	0.231	0.212	0.030	0.004	0.004												
**LSW**	0.161	0.192	0.186	0.246	0.264	0.241	0.046	0.004	0.008	0.009											
**MAU**	0.272	0.082	0.198	0.112	0.100	0.089	0.162	0.225	0.190	0.204	0.229										
**TIN**	0.174	0.202	0.187	0.252	0.271	0.249	0.055	0.020	0.022	0.022	0.015	0.236									
**MSC**	0.152	0.175	0.167	0.226	0.243	0.222	0.030	0.006	**0.001**	0.002	0.004	0.211	0.018								
**MSG**	0.137	0.153	0.153	0.203	0.218	0.198	0.024	0.009	**0.001**	0.003	0.007	0.190	0.022	**-0.002**							
**MSK**	0.189	0.094	0.153	0.127	0.123	0.112	0.096	0.143	0.113	0.125	0.137	0.128	0.147	0.122	0.103						
**MSV**	0.152	0.167	0.163	0.219	0.235	0.215	0.034	0.007	0.004	0.003	0.007	0.205	0.022	**0.001**	**0.001**	0.121					
**PIG**	0.482	0.461	0.486	0.473	0.506	0.484	0.354	0.382	0.365	0.371	0.444	0.468	0.443	0.441	0.417	0.410	0.435				
**OTT**	0.194	0.128	0.175	0.181	0.200	0.179	0.083	0.138	0.109	0.122	0.135	0.166	0.140	0.122	0.103	0.111	0.117	0.469			
**TRA**	0.499	0.551	0.518	0.521	0.555	0.538	0.377	0.397	0.388	0.389	0.487	0.534	0.492	0.479	0.453	0.435	0.477	0.709	0.571		
**TRE**	0.320	0.079	0.222	0.064	0.048	0.035	0.199	0.265	0.230	0.245	0.276	0.111	0.285	0.257	0.233	0.143	0.250	0.521	0.215	0.567	
**YAM**	0.232	0.260	0.246	0.295	0.312	0.295	0.164	0.203	0.179	0.191	0.210	0.288	0.222	0.205	0.187	0.216	0.200	0.520	0.240	0.574	0.330

Table S3 Description of the major models tested in this study.

Model 1 to 17 described the major model tested without mortality rate. These models were tested with varying population size of the 3 descendent population (N= 400/600/800/1600).

Model 18 to 23 are the equivalent of model 9 to 11 with varying mortality rate (column Death rate) and with varying migration rate from LDM to SLR.SRC = Source population used for stocking. Given the shared patterns of ancestry between the population used for stocking (i.e. Chautauqua, Joseph and Tremblant lake) individuals were merged together and modelled as a single unit. Given that no sign of ancestry from Pigeon lake was find in the Saint Lawrence (SLR) or the Lac des Deux-Montagnes (LDM) it was not modelled here.

Based on our observation of higher admixture in LDM than in SLR, the migration rate (m) was twice higher in LDM than in SLR.

name	N (Size)	Death rate	m SRC → SLR	m SRC → LDM	m SLR → LDM	m LDM → SLR
model01	400/600/800/1600	0	0.000001	0.000002	0.0005	0.001
model02	400/600/800/1600	0	0.00001	0.00002	0.0005	0.001
model03	400/600/800/1600	0	0.0001	0.000015	0.0005	0.001
model04	400/600/800/1600	0	0.001	0.00015	0.0005	0.001
model05	400/600/800/1600	0	0.01	0.0015	0.0005	0.001
model06	400/600/800/1600	0	0.005	0.0075	0.0005	0.001
model07	400/600/800/1600	0	0.0033	0.005	0.0005	0.001
model08	400/600/800/1600	0	0.0025	0.00375	0.0005	0.001
model09	400/600/800/1600	0	0.005	0.0075	0.0003	0.00015
model10	400/600/800/1600	0	0.0033	0.005	0.0003	0.00015
model11	400/600/800/1600	0	0.0025	0.00375	0.0003	0.00015
model12	400/600/800/1600	0	0.1	0.15	0.0005	0.001
model13	400/600/800/1600	0	0.05	0.075	0.0005	0.001
model14	400/600/800/1600	0	0.025	0.0375	0.0005	0.001
model15	400/600/800/1600	0	0.15	0.225	0.0005	0.001
model16	400/600/800/1600	0	0.2	0.3	0.0005	0.001
model17	400/600/800/1600	0	0.015	0.0025	0.0005	0.001
model18	400/600/800/1600	50/100/200/400	0.005	0.0075	0.0005	0.001
model19	400/600/800/1600	50/100/200/400	0.0033	0.005	0.0005	0.001
model20	400/600/800/1600	50/100/200/400	0.0025	0.00375	0.0005	0.001
model21	400/600/800/1600	50/100/200/400	0.005	0.0075	0.0003	0.00015
model22	400/600/800/1600	50/100/200/400	0.0033	0.005	0.0003	0.00015
model23	400/600/800/1600	50/100/200/400	0.0025	0.00375	0.0003	0.00015

Supplementary Methods and Results

1. Measurement of the symmetry of migration using coalescent simulations and ABC

Methods

Migration rate (*m)* was measured using coalescent simulations combined with an ABC procedure for parameter estimation. The objective was to determine if sample sites from the St. Lawrence that displayed weak level of genetic differentiation (e.g. Fst < 0.01) were connected by gene flow or if this was due to populations with large Ne evolving independently (i.e. with very low gene flow).

a) model and coalescent simulations

The demographic history consists in a set of two subpopulations exchanging migrants in a stepping stone model. Each subpopulation is of size $N_{upstream}$ and $N_{downstream}$ and migrants are exchanged at a rate $M_{ij} = 4N_0m_{ij}$ at each generation. Here m_{ij} is the fraction of the subpopulation *i* made of migrants of each generation from sub-population *j*. Migration is modelled randomly and uniformly for each of the upstream and downstream sub-population respectively. Prior for effective population size were uniformly distributed on the interval [0 – 500,000] and sampled independently for each sub-population. Prior for M were sampled initially, randomly on the interval [0-40]. Prior were generated with a modified version of priorgen (Ross-Ibarra et al., 2008), recoded in Python and modified to implement this new model.

Coalescent simulations were performed in msnsam, a modified version of ms allowing for variable sample size among loci (Hudson, 2002; Ross-Ibarra et al., 2008). Here, we set the size of the reference population Nref to 50,000 (the choice of Nref is arbitrary and used as a scaling factor, without impact on the estimated parameter.

We set the mutation rate μ to $1x10^{-8}$ bp/generation, following many others (e.g. (Barrio et al., 2016; Rougeux, Bernatchez, & Gagnaire, 2017; Tine et al., 2014). While this choice may seem arbitrary, it cancels out when ratio of demographic parameters (*M* and *N*) are used and is of minimal concern here. Therefore, we set $\theta = 4N_{ref}\,\mu L$ to 0.16. Here L is the length of the RAD loci (80 pb).

A first round of ABC parameters estimation indicated that the upper bound of the prior distribution was attained at a value of 40 (see results below) while all other parameters were very well estimated. We run another round using a prior ranging between [0-80]. Since all other parameters were very well estimated, we fixed a narrow prior based on the minimum and maximum value of the posterior distribution from the previous estimation.

b) ABC parameters estimation

One million coalescent simulations were generated and associated summary statistics were computed using mscalc (Camille Roux et al., 2011)R for each small sequence. The summary statistics are the same as in (Rougemont & Bernatchez, 2018). These include the mean and standard variations of : the nucleotide diversity π (Tajima, 1989), the net divergence between the two populations (D_A), the total divergence between populations (D_{XY}) (Nei & Kumar, 2000), the between population differentiation (Fst) value, the number of fixed differences between populations (Sf); the number of polymorphic sites private to each population (Sx*pop1* and Sx*pop2*) and the number of shared polymorphic sites (Ss); and last the Pearson's R2 coefficient of correlation in *π* between the two populations. These statistics represent a panel of commonly used summary indexes in ABC (Fagundes et al., 2007; Ross-Ibarra et al., 2008; C. Roux, Tsagkogeorga, Bierne, & Galtier, 2013; Camille Roux et al., 2016).

Parameters estimation was performed using the "abc" package (Csilléry, François, & Blum, 2012). We used a logit transformation of the parameters and a tolerance of 0.001. The posterior probabilities of parameter values were then estimated using the neural network procedure with nonlinear regressions of the parameters on the summary statistics using 50 feed-forwards neural networks and 15 hidden layers. All scripts to reproduce the results are available on github.

c) Choice of populations

The demographic model was tested on two samples located on the St. Lawrence at two extremities of our sampling range. Namely we chose the Lake St. Pierre as the downstream site and lake St. François as our upstream site. Although more upstream populations were available (TIN, Thousand Islands or LSW, St. Lawrence Lake) these sites were also separated by impassable barriers to gene flow (dams) in the upstream direction at least. Such dams would have certainly obscured our inference.

d) Results

<u>First round of Parameter estimates:</u>

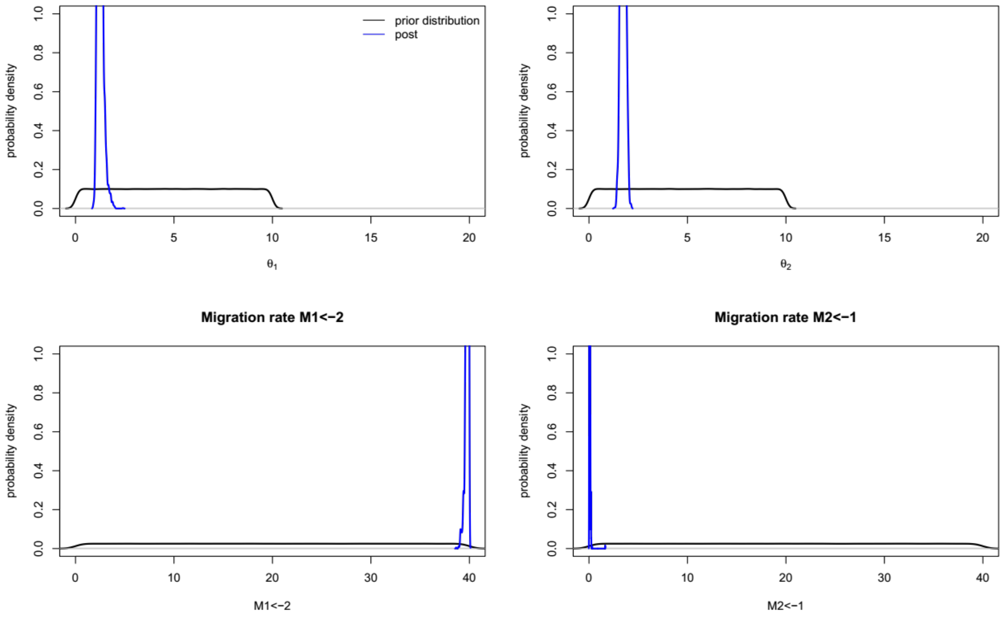

Figure S5: **Posterior distribution of parameter estimates under the demographic model.**
Theta 1 = scaled effective population size of the downstream population (Lake St. Pierre)
Theta 2 = scaled effective population size of the upstream population (Lake St. François)
Migration rate $M_{1\leftarrow 2}$ migration from upstream to downstream ($M_{ij} = 4N_0m_{ij}$)
Migration rate $M_{2\leftarrow 1}$ migration from downstream to upstream.
All values are scaled (in coalescent units).
The black line represents the prior distribution and the blue line represents the posterior distribution obtained after neural-network regression of the 1,000 retained simulations closest to the observed data.

Given that the maximum value of the prior on $M_{1\leftarrow 2}$ is reached in this model we performed a new set of 1 million coalescent simulations but using a larger bound on the uniform prior. We used M = [0 – 80]. Since the remaining parameters were very well estimated, we use the [min-max] value of the posterior of N1, N2, M2 in these simulations. This was done in order to reduce the number of "uninformative" simulations generated by too large prior as this first round indicated which of the simulation were informative.

<u>Second round of Parameter estimates:</u>

The results for the second round indicated similar estimates for N1 and N2 as in the first round, as expected from their very narrow minimal and maximal posterior distributions. Values for $M_{2\leftarrow 1}$ was closer to its previously inferred maximal distribution (median = 1.99 [95%CI = 1.88 – 2.00], Figure S6 below).
The value for $M_{1\leftarrow 2}$ again reached the upper bound of M = 80 [95CI = 76.80 – 80]. This would translate into a high number of migrants (i.e. > 22).

e) Discussion

On the first round of parameters estimation, we note that the upper bound of the prior for $M_{1\leftarrow 2}$ is reached. Given the hypothesized performed above (Nref = 5e4) we obtained *m = 0.002 [95%CI = 39.9-40].* A biologically meaningful number is the total number of migrant each generation. The median value of N1 given the posterior distribution is 1.2284*5e4 = 61,420 [95%CI = 1.0391 – 1.5922]*5e4.
This give 12.284 migrants each generation in the downstream populations. [95%CI = 10.4 – 16] migrants.

Conversely, in the upstream population we have a median value of $M_{2\leftarrow 1}$ = 6.671e-02 [95%CI = 1.824e-02 – 1.398e-01] and a median of N_2 = 1.72*5e4 [95%CI = 1.5127 – 1.9697]*5e4.
This translate into 0.028 [95%CI = 0.007 – 0.068] migrants.

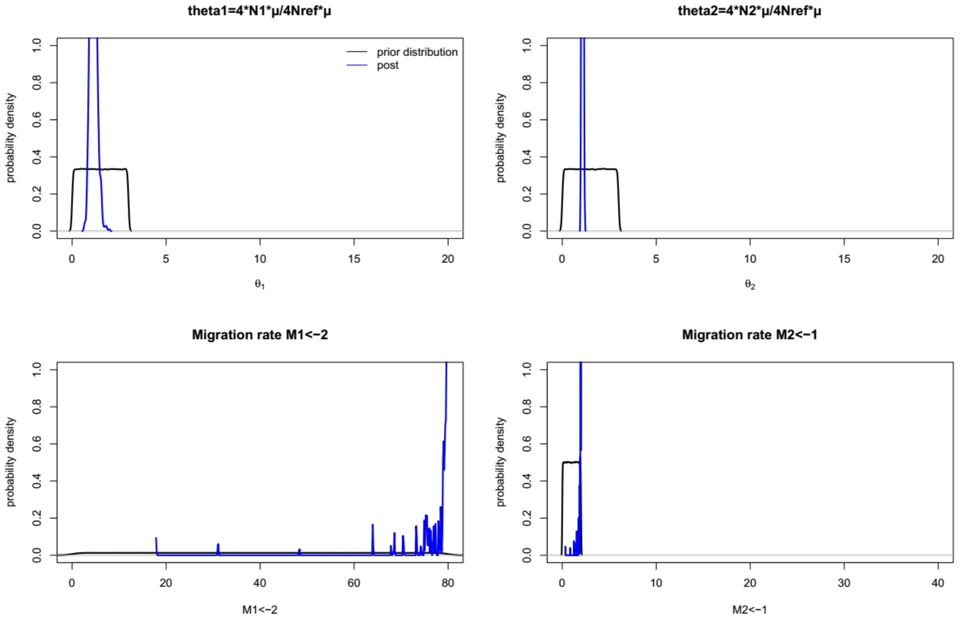

Figure S6: **Parameter estimates under the demographic model using constrained prior on N1, N2 and M_{21} based on the previously inferred parameters.** Prior on $M_{1\leftarrow2}$ was set to twice its previous value.

The second round of parameter estimation also provides unambiguous evidence for higher downstream directed migration than the reverse. Indeed, the ratio $M_{1\leftarrow2}$/ $M_{2\leftarrow1}$ indicates that a 40 times higher downstream directed migration. We therefore suggest that setting the upper bound to higher value would lead to still higher estimates, suggesting that overall, the model may converge to a model of panmixia.
From this inference we conclude that 1) populations are highly connected, 2) connectivity is due to downstream biased dispersal, as expected from theory and 3) the population show only minor deviation from panmixia.
From a conservation perspective, we suggest that maintaining high connectivity among localities is the best way to maintain genetic diversity.

2. Measurement of the divergence time under a model of Strict Isolation

a) Motivation

Observed patterns of genetic differentiation (Fst) were strong between several isolated lakes. A closer look at the data indicates low levels of polymorphism, and that most of the variation was still shared between lakes. This raise the trivial question of whether strong differentiation and strong structure are due to a long time of divergence or other processes (bottlenecks, small population size). Addressing this question allows formulating further working hypothesis related to the probabilities of accumulating genetic incompatibilities or to display outbreeding depression or not.

Here, we tackle this question by studying a model of evolutionary divergence between the two lakes initially used for stocking, namely Pigeon lake and Chautauqua. In this case, the lakes are separate by a large distance (see Fig 1) and we make the assumption that there is no ongoing gene flow possible. Therefore, we used a simplified model of strict isolation to estimate the divergence time of these two populations. It is obvious that any initial gene flow should delay the speed of divergence.

In a second analysis, we wanted to verify if the St. Lawrence have diverged more or less at the same time than the two initially compared lakes. To do so we randomly picked sites from within the St. Lawrence (we chose sites with approximately similar numbers of individuals) and compared them to the lakes. We hypothesized that if the St. Lawrence (or its tributaries) diverged at the same time than other lakes, this would provide support for a single divergence event, perhaps due to recolonization of the area after some geological events. Under this scenario, the colonization of the smallest lake by few individuals can explain their lower genetic diversity.

b) Model and coalescent simulations

The strict isolation (SI) model was used for coalescent simulations. This model is characterized by the instantaneous split (at time Tsplit) of an ancestral population of size N_{anc} into two daughter population of size N_1 and N_2. There is no subsequent gene flow between the population. Population size can vary at the split time so that the daughter population may undergo a bottleneck at this particular moment.
The same procedure as above was used: Prior for effective population size were uniformly distributed on the interval [0 – 1,000,000] individuals. These were sampled independently for each sub-population and for the ancestral populations.

Prior for Tsplit was sampled on the interval [0-6,000,000] generations. Prior were generated with a modified version of priorgen (Ross-Ibarra, 2008), recoded in Python.

Coalescent simulations were performed in msnsam with Nref set to 50,000.

We set the mutation rate μ to $1x10^{-8}$ bp/generation, following many others (e.g. Barrio et al. 2016; Tine et al. 2014; Rougeux et al. 2017). Therefore we set $\theta = 4N_{ref}\ \mu L$ to 0.16. Here L is the length of the RAD loci (80 pb).
The impact of arbitrary chosen mutation rate is discussed recently in (Brock & Wagner, 2018).
Here, to verify how different mutations rate would affect our conclusions, we also run the simulation pipeline by fixing a mutation rate 5 times lower (i.e. $\mu = 2x10^{-9}$ bp/generation translating into theta = 0.032) corresponding to the lowest estimates of μ in vertebrates, namely the Atlantic herring (Feng et al., 2017). An intermediate mutation rate produce similar results and we provide below only the estimates for these two extremes.

The analysis was replicated 5 times but comparing one randomly chosen site from within the St. Lawrence to either Pigeon lake or Chautauqua lake. This allowed us to test if all populations approximately have split simultaneously.

c) ABC parameter estimation

One million coalescent simulations were generated and associated summary statistics were computed using mscalc (Roux et al. 2011) for each small sequence. The summary statistics are the same as in Rougemont & Bernatchez (2018) and are described above. The exact same procedure for parameter estimation was used. All scripts to exactly reproduce the results are available on github.

d) Results

1) Comparison between stocking sources: Chautauqua and Pigeon lake

The posterior distribution of parameters was very well differentiated from the prior distribution for the divergence time and ancestral population size parameter, resulting in narrow credible intervals (Fig S7). The descending population sizes were less accurately estimated and we do not interpret them.

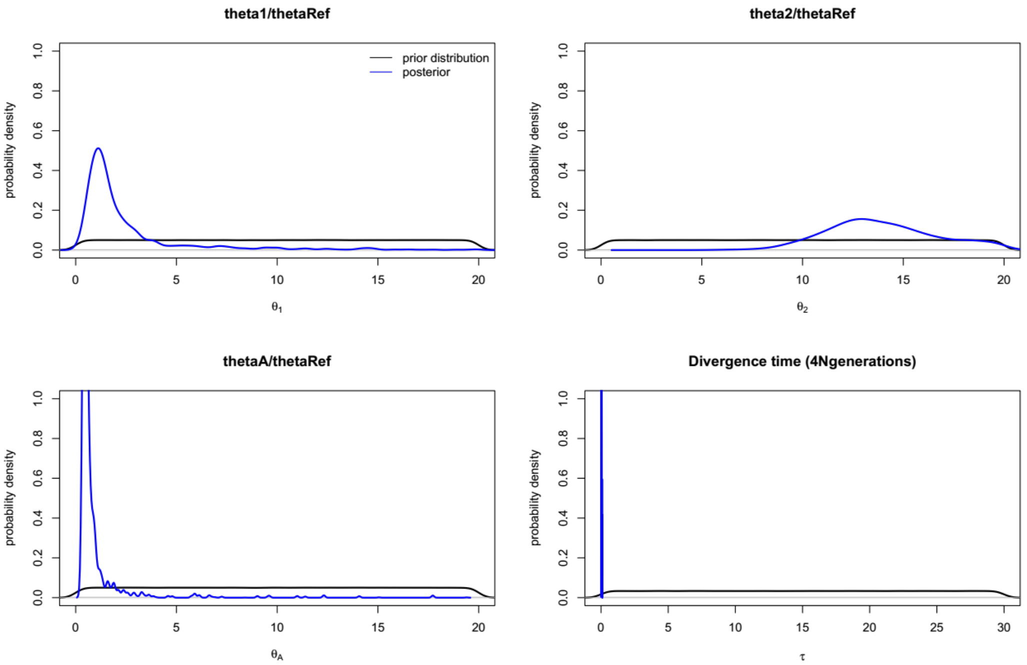

Figure S7: **Posterior distribution of parameter estimates under a model of strict isolation between Chautauqua and Pigeon lake.**
Θ1 = scaled effective population size of Pigeon lake (4N1μ/NRefμ)
Θ2 = scaled effective population size of Chautauqua lake (4N2μ/Nrefμ)
ΘA = scaled ancestral effective population size (4N1μ/Nrefμ)
τ = Tsplit/(4Nref) scaled divergence time.
Prior distribution of the parameter is displayed in black and the posterior distribution is in blue.

In this case, under the hypothetical mutation rate, we obtain a median divergence time of 22,000 years [95%CI = 10,000 – 45,000] years.

To test the influence of the mutation rate, we run the coalescent simulation with θref = 0.032.
Parameter estimates were not informative for the daughter population but only for the ancestral population and for the divergence time (Figure S8):

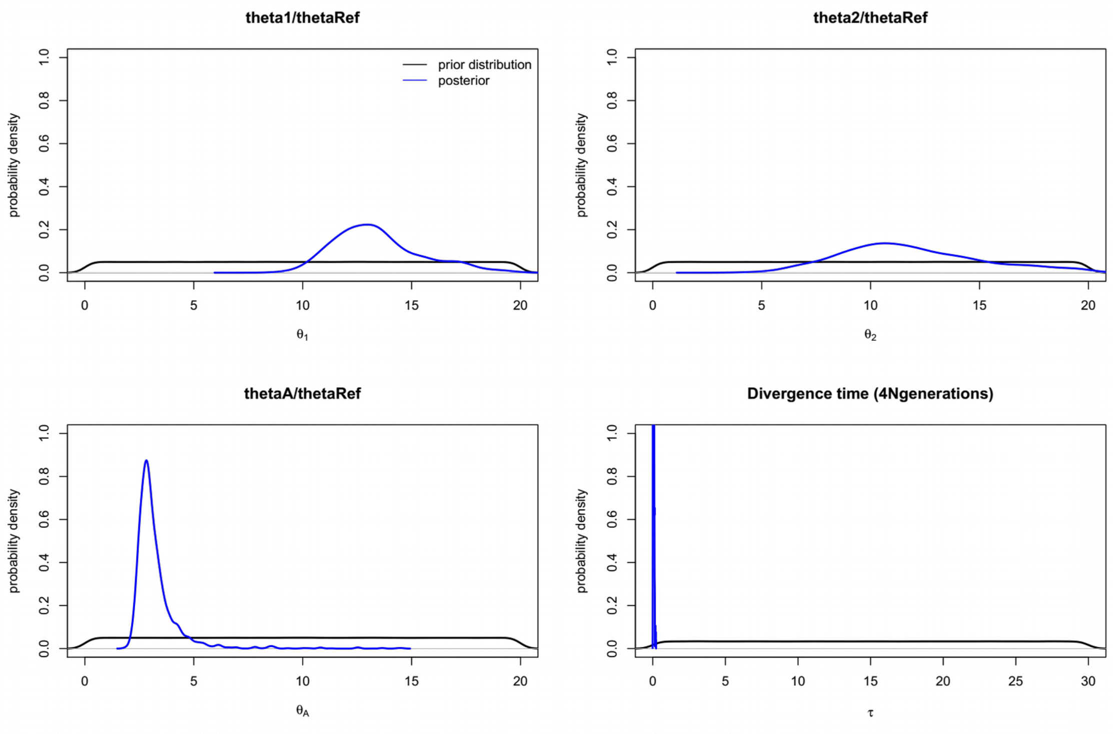

Figure S8: **Estimated divergence time using a 5 times smaller mutation rate in the coalescent simulations.**
Θ1 = scaled effective population size of Pigeon lake (4N1μ/NRefμ)
Θ2 = scaled effective population size of Chautauqua lake (4N2μ/Nrefμ)
ΘA = scaled ancestral effective population size (4N1μ/Nrefμ)
τ = Tsplit/(4Nref) scaled divergence time.
Prior distribution of the parameter is displayed in black and the posterior distribution is in blue.

In this case, converting the divergence time yield a median Tsplit = 26,000 [7,000 – 70, 000] years.

Each analyses using sampling sites from the St. Lawrence yielded similarly very accurate posterior distribution of the split time (τ) with a minimum median value of 10,700 years [CI = 4,550 – 17000] and a maximum value of 19,040 [95%CI = 8,800 – 29,610] years.

The posterior distribution of parameter value for each compared population is provided in the table below. The posterior distribution of the parameter relative to the prior is also provided in the Figures below for each pairs of populations.

Table S4: Parameters estimates for each pairs of compared sites. Abbreviation of the sampling site are given in table 1. N1,N2 and Na are the respective effective population size of population 1 population 2 and ancestral population. Tsplit is the time of divergence. 95% Credible Intervals are given in brackets.

pop1	pop2	N1 [95%CI]	N2 [95%CI]	Na [95%CI]	Tsplit [95%CI]
LSF	CHQ	221425 [82840 -- 877540]	210435 [84420 -- 902680]	22955 [13765 -- 173305]	15400 [6500 -- 24300]
ONT	CHQ	72543 [23906 -- 624831]	682064 [459594 -- 974402]	26174 [15772 -- 192508]	22925 [10097 -- 45688]
LSP	CHQ	481555 [259325 -- 956745]	162190 [73725 -- 738125]	19700 [13540 -- 124335]	17500 [8500 -- 25800]
ONT	LSF	198185 [88606 -- 807925]	485464 [259816 -- 945909]	11394 [7359 -- 90519]	17967 [10429 -- 25575]
ONT	MSG	291165 [78978 -- 906731]	883355 [597543 -- 997576]	20516 [13076 -- 144742]	18385 [6904 -- 31455]
LSL	CHQ	415680 [176285 -- 960540]	72280 [25755 -- 631380]	19400 [12390 -- 120585]	19300 [9400 -- 31200]
TRA	LSF	24834 [11879 -- 124296]	690383 [454062 -- 975493]	12240 [9093 -- 57088]	15975 [8285 -- 25267]
MAU	LSF	108610 [38430 -- 798615]	268575 [100410 -- 916210]	30085 [18850 -- 290270]	27200 [12700 -- 42300]
MAU	LSP	66440 [14770 -- 758395]	342890 [107870 -- 963360]	26085 [18380 -- 173160]	13700 [7700 -- 20900]

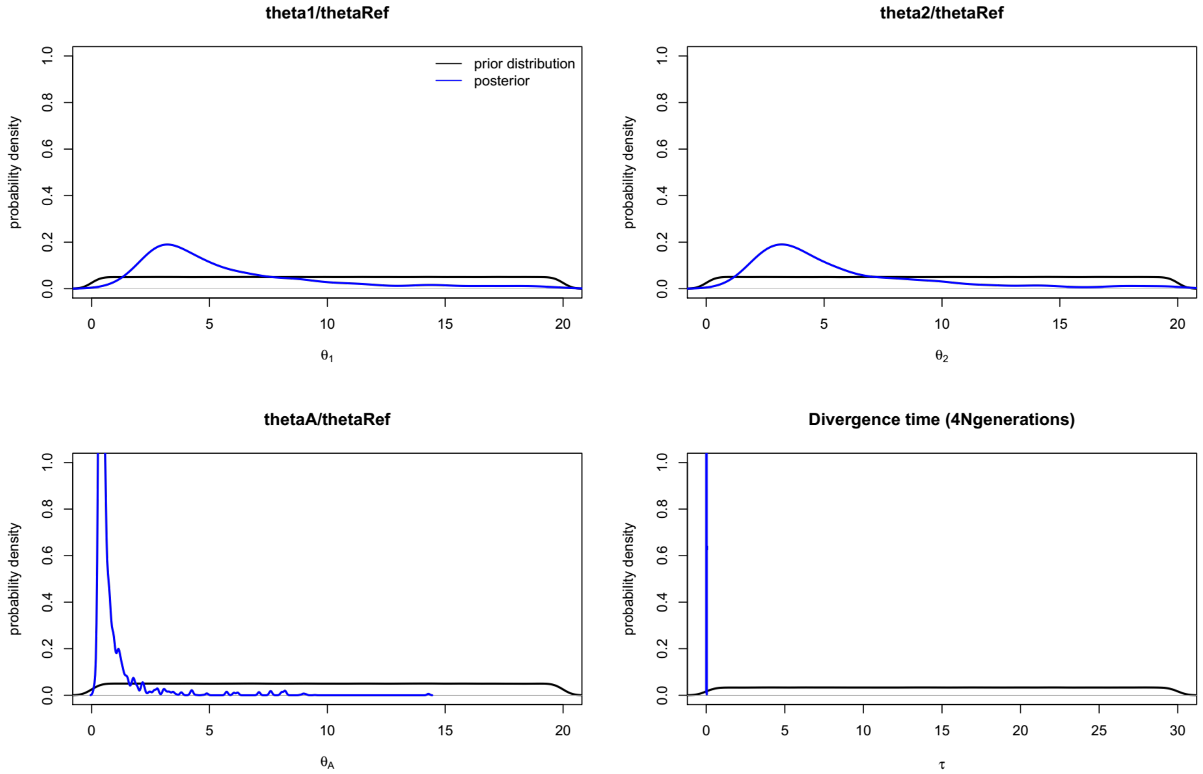

***Figure S9*: Estimated divergence time between St. Francis lake and Chautauqua lake.**
Θ1 = scaled effective population size of St. Francis lake (4N1μ/NRefμ)
Θ2 = scaled effective population size of Chautauqua lake (4N2μ/Nrefμ)
ΘA = scaled ancestral effective population size (4N1μ/Nrefμ)
τ = Tsplit/(4Nref) scaled divergence time.
Prior distribution of the parameter is displayed in black and the posterior distribution is in blue.

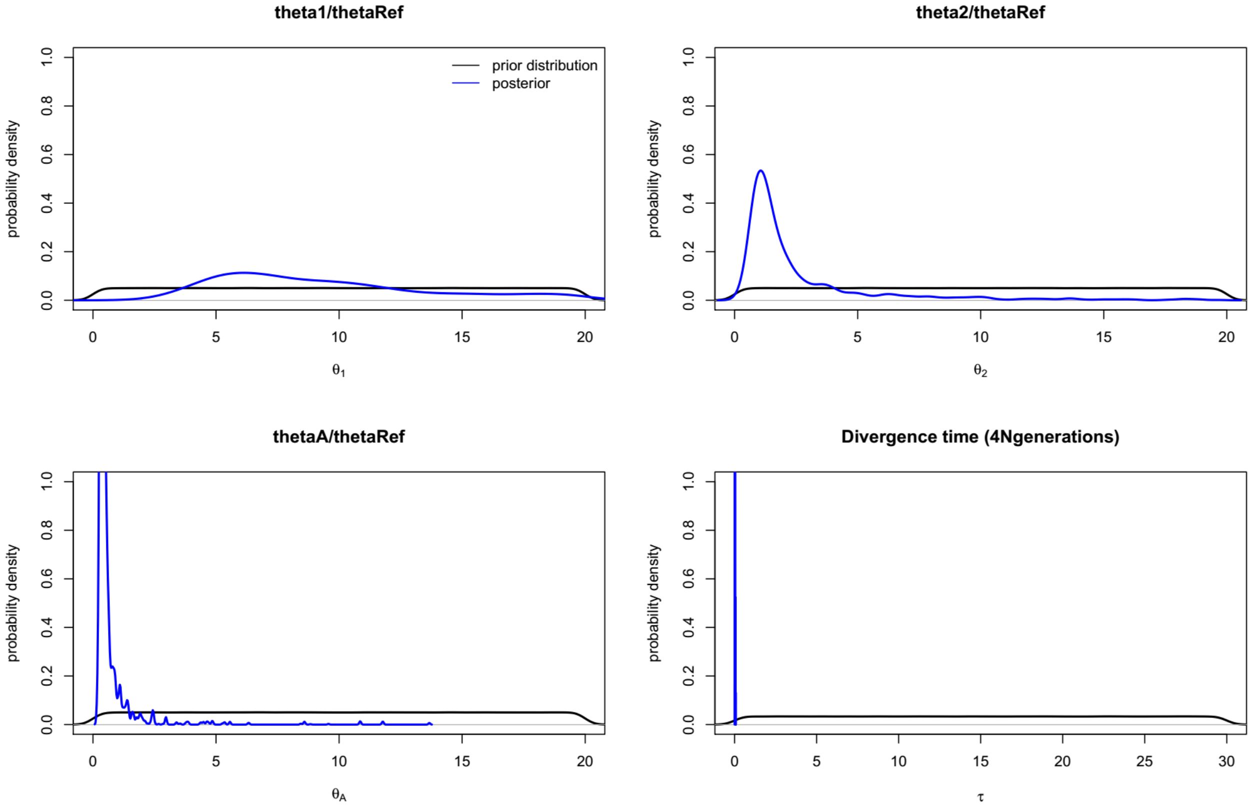

Figure S10: **Estimated divergence time between St. Louis lake and Chautauqua lake.**
Θ1 = scaled effective population size of St. Louis lake (4N1μ/NRefμ)
Θ2 = scaled effective population size of Chautauqua lake (4N2μ/Nrefμ)
ΘA = scaled ancestral effective population size (4N1μ/Nrefμ)
τ = Tsplit/(4Nref) scaled divergence time.
Prior distribution of the parameter is displayed in black and the posterior distribution is in blue.

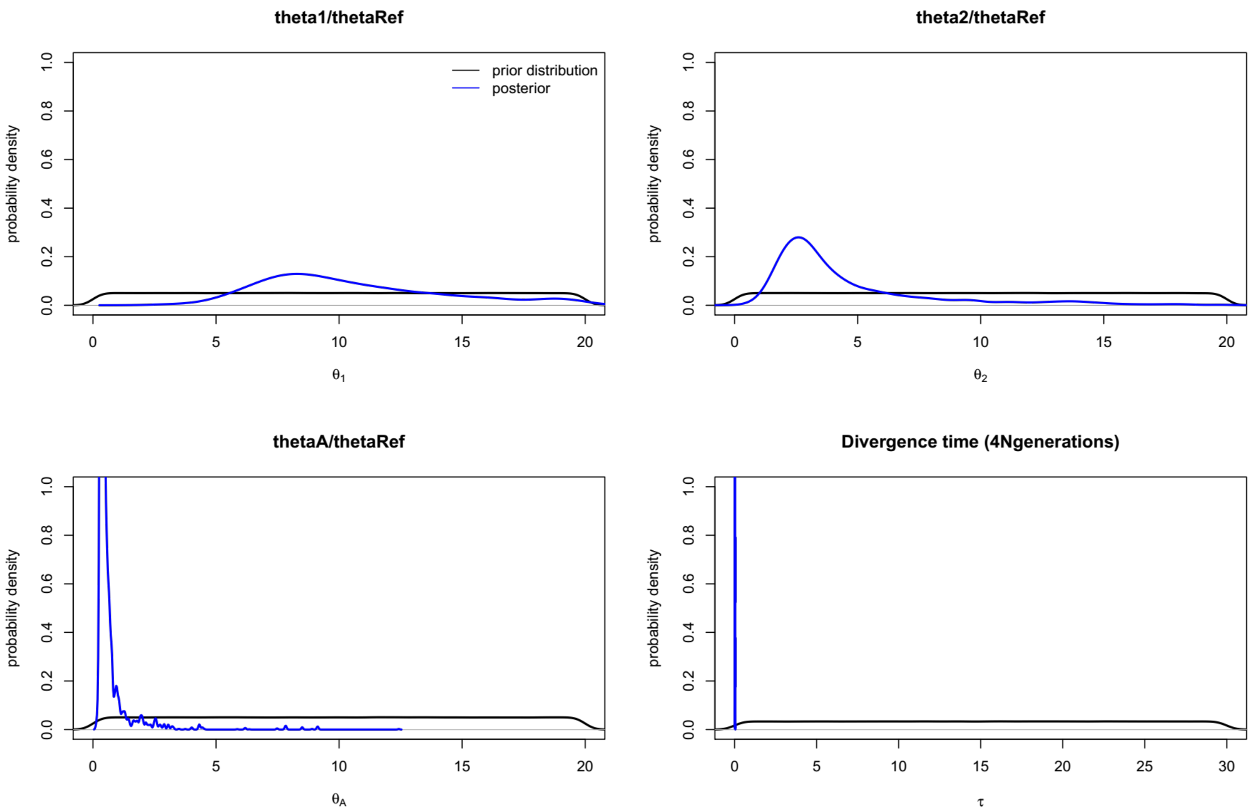

Figure S11: **Estimated divergence time between St. Pierre lake and Chautauqua lake.**
Θ1 = scaled effective population size of St. Pierre lake (4N1μ/NRefμ)
Θ2 = scaled effective population size of Chautauqua lake (4N2μ/Nrefμ)
ΘA = scaled ancestral effective population size (4N1μ/Nrefμ)
τ = Tsplit/(4Nref) scaled divergence time.
Prior distribution of the parameter is displayed in black and the posterior distribution is in blue.

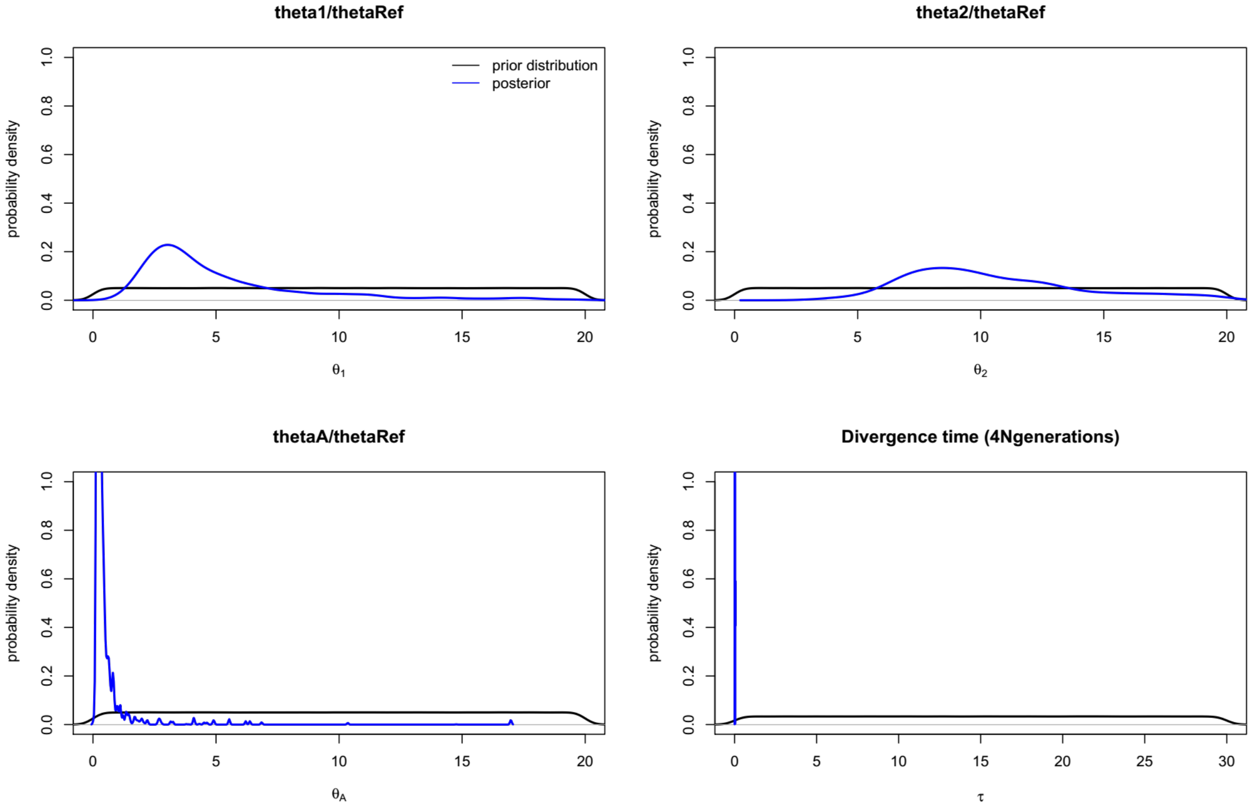

***Figure S12*: Estimated divergence time between Pigeon lake and St. Francis lake.**
Θ1 = scaled effective population size of Pigeon lake (4N1μ/NRefμ)
Θ2 = scaled effective population size of St. Francis lake (4N2μ/Nrefμ)
ΘA = scaled ancestral effective population size (4N1μ/Nrefμ)
τ = Tsplit/(4Nref) scaled divergence time.
Prior distribution of the parameter is displayed in black and the posterior distribution is in blue.

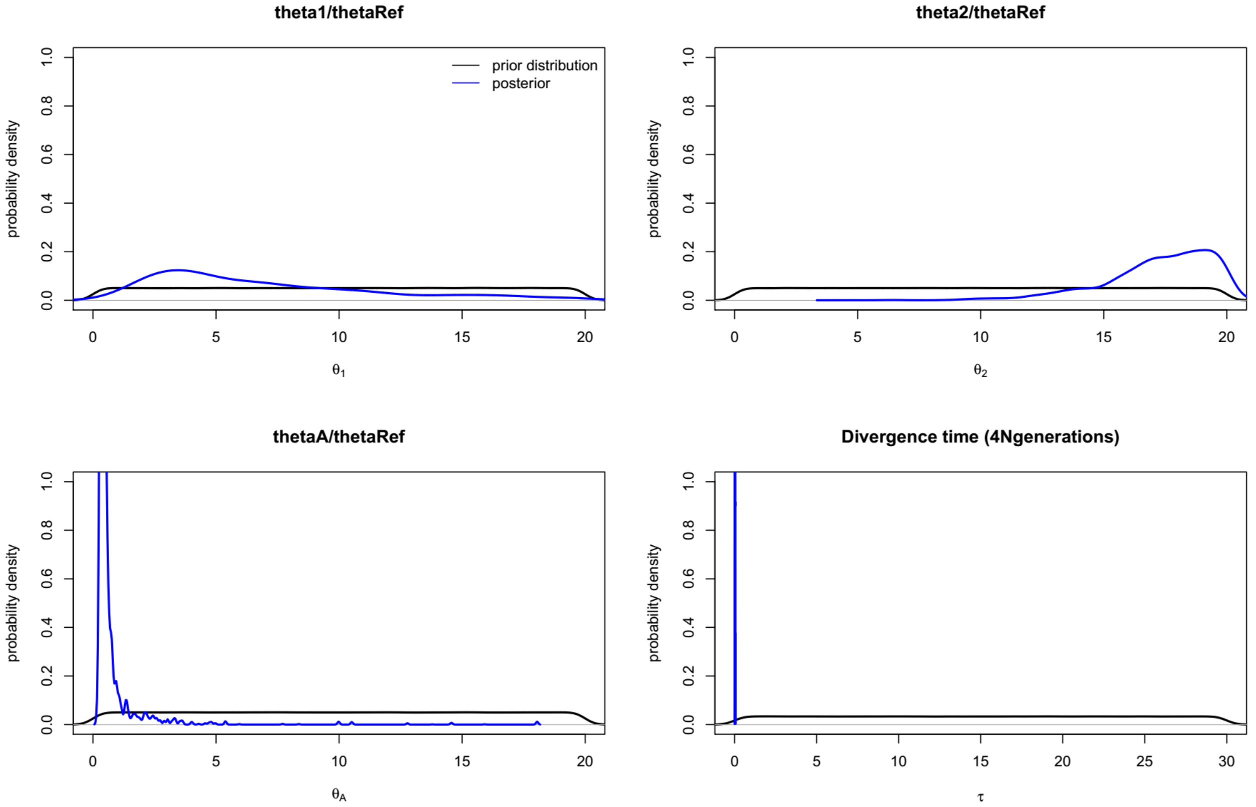

***Figure S13*: Estimated divergence time between Pigeon lake and Montreal sorel.**
Θ1 = scaled effective population size of Pigeon lake (4N1µ/NRefµ)
Θ2 = scaled effective population size of Montreal sorel (4N2µ/Nrefµ)
ΘA = scaled ancestral effective population size (4N1µ/Nrefµ)
τ = Tsplit/(4Nref) scaled divergence time.
Prior distribution of the parameter is displayed in black and the posterior distribution is in blue.

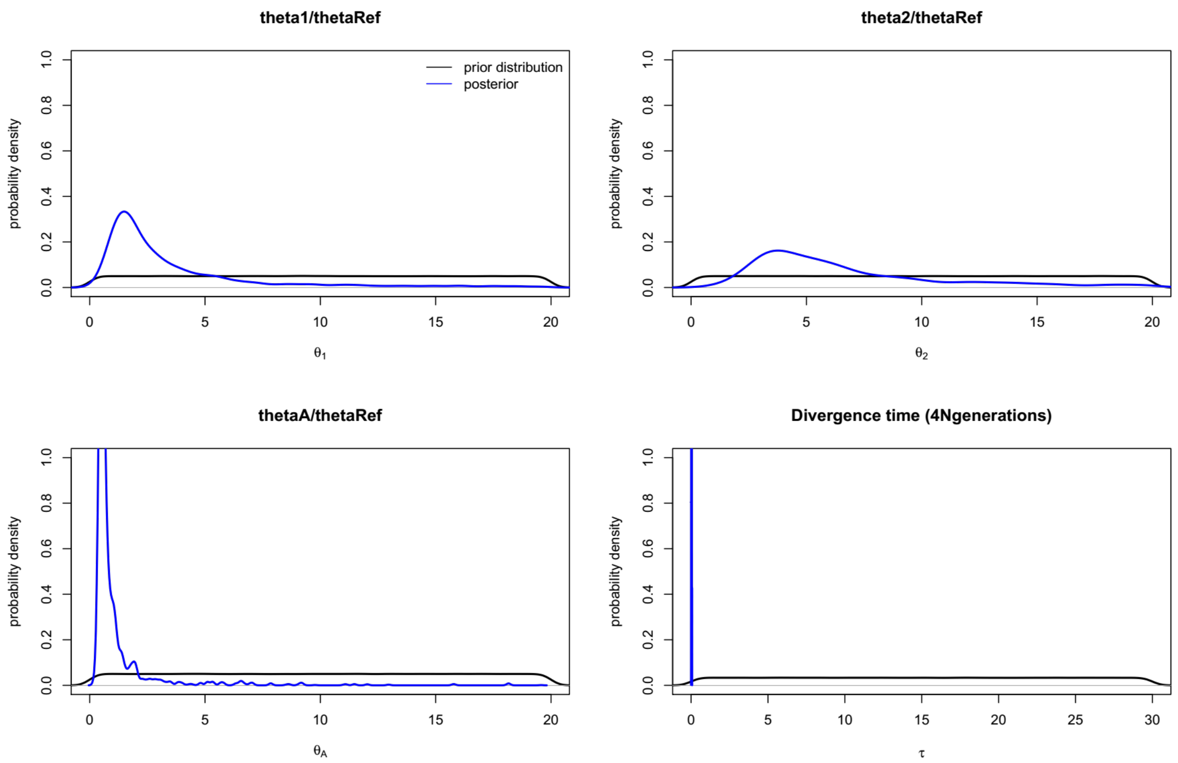

Figure S14: **Estimated divergence time between St. Maurice river and St. Francis lake.**
Θ1 = scaled effective population size of St. Maurice lake (4N1μ/NRefμ)
Θ2 = scaled effective population size of St. Francis lake (4N2μ/Nrefμ)
ΘA = scaled ancestral effective population size (4N1μ/Nrefμ)
τ = Tsplit/(4Nref) scaled divergence time.
Prior distribution of the parameter is displayed in black and the posterior distribution is in blue.

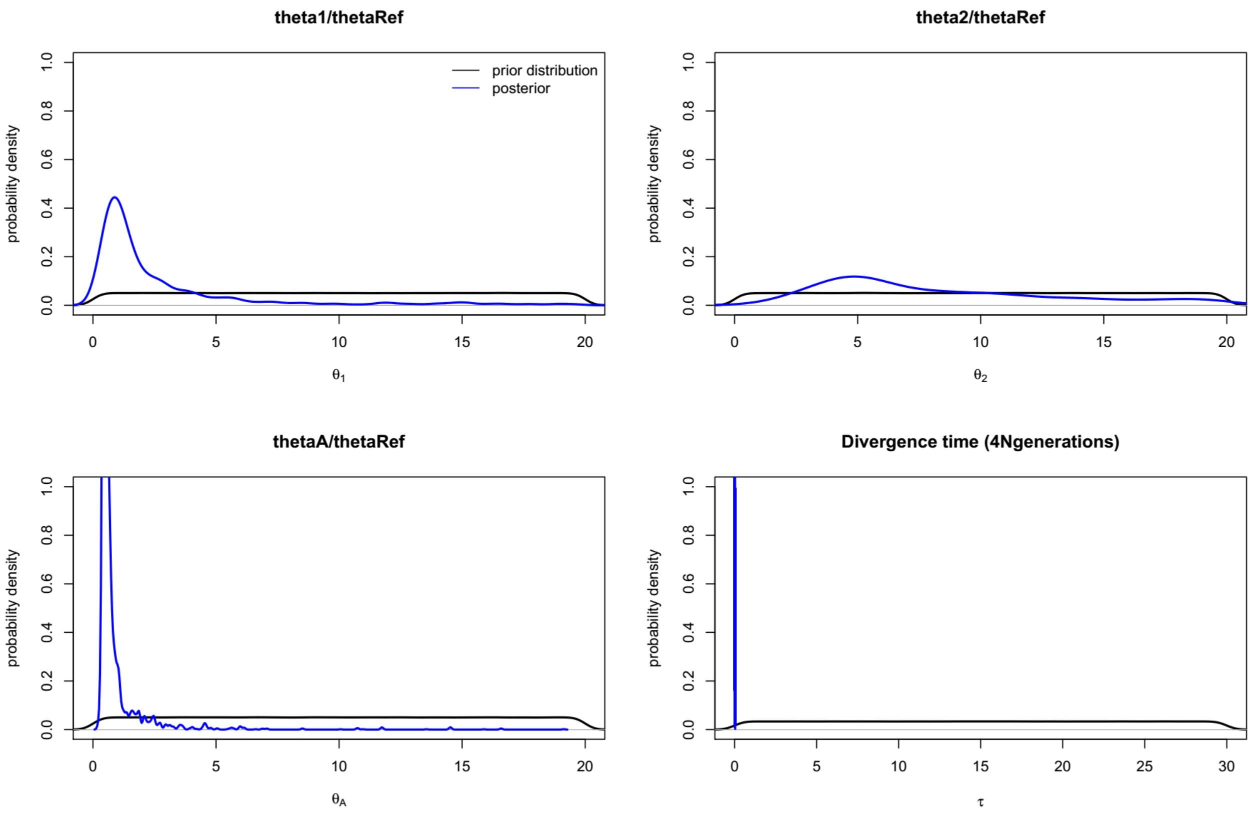

***Figure S15: E*stimated divergence time between st. Maurice river and St. Pierre lake.**
Θ1 = scaled effective population size of St. Maurice river (4N1µ/NRefµ)
Θ2 = scaled effective population size of St. Pierre lake (4N2µ/Nrefµ)
ΘA = scaled ancestral effective population size (4N1µ/Nrefµ)
τ = Tsplit/(4Nref) scaled divergence time.
Prior distribution of the parameter is displayed in black and the posterior distribution is in blue.

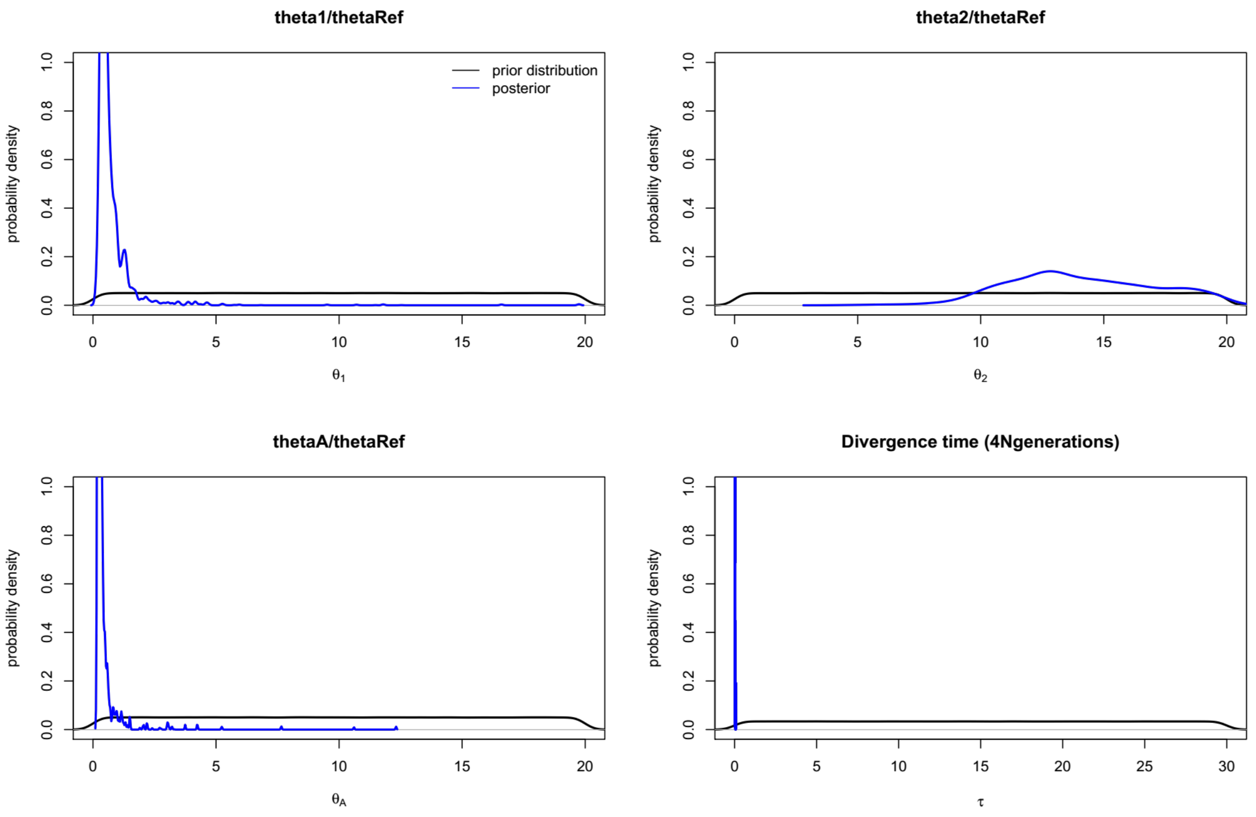

Figure S16: **Estimated divergence time between st. Maurice river and St. Pierre lake.**
Θ1 = scaled effective population size of St. Maurice river (4N1μ/NRefμ)
Θ2 = scaled effective population size of St. Pierre ake (4N2μ/Nrefμ)
ΘA = scaled ancestral effective population size (4N1μ/Nrefμ)
τ = Tsplit/(4Nref) scaled divergence time.
Prior distribution of the parameter is displayed in black and the posterior distribution is in blue.

e) Discussion & limits

Estimates of divergence time were congruent across each pair of population tested. On the contrary we failed to estimate contemporary population sizes (N1 and N2) in most of the cases.

These estimates are based on assumption regarding the mutation rate that affect more or less our biological estimates. Reassuringly, estimates performed using two different mutation rate still resulted in overlapping 95% credible intervals. Regardless of the mutation rate that affect all our models in the same way, it is likely that all populations have started to diverged simultaneously, for instance at the end of the last glacial event, when the many meters of ice started to melt. At this time, individuals may have been able to invade the water from Quebec, resulting in the establishment (and divergence) of these recent populations.

References:

Barrio, A. M., Lamichhaney, S., Fan, G., Rafati, N., Pettersson, M., Zhang, H., … Andersson, L. (2016). The genetic basis for ecological adaptation of the Atlantic herring revealed by genome sequencing. *ELife*, *5*, e12081. https://doi.org/10.7554/eLife.12081

Brock, C. D., & Wagner, C. E. (2018). The smelly path to sympatric speciation? *Molecular Ecology*, *27*(21), 4153-4156. https://doi.org/10.1111/mec.14845

Csilléry, K., François, O., & Blum, M. G. B. (2012). abc: an R package for approximate Bayesian computation (ABC). *Methods in Ecology and Evolution*, *3*(3), 475-479. https://doi.org/10.1111/j.2041-210X.2011.00179.x

Fagundes, N. J. R., Ray, N., Beaumont, M., Neuenschwander, S., Salzano, F. M., Bonatto, S. L., & Excoffier, L. (2007). Statistical evaluation of alternative models of human evolution. *Proceedings of the National Academy of Sciences*, *104*(45), 17614-17619. https://doi.org/10.1073/pnas.0708280104

Feng, C., Pettersson, M., Lamichhaney, S., Rubin, C.-J., Rafati, N., Casini, M., … Andersson, L. (2017). Moderate nucleotide diversity in the Atlantic herring is associated with a low mutation rate. *eLife*, *6*, e23907. https://doi.org/10.7554/eLife.23907

Hudson, R. R. (2002). Generating samples under a Wright-Fisher neutral model of genetic variation. *Bioinformatics (Oxford, England)*, *18*(2), 337-338.

Nei, M., Kumar, S. (2000). Molecular Evolution and Phylogenetics. Oxford University Press: New York.

Ross-Ibarra, J., Wright, S. I., Foxe, J. P., Kawabe, A., DeRose-Wilson, L., Gos, G., … Gaut, B. S. (2008). Patterns of Polymorphism and Demographic History in Natural Populations of Arabidopsis lyrata. *PLoS ONE*, *3*(6), e2411. https://doi.org/10.1371/journal.pone.0002411

Rougemont, Q., & Bernatchez, L. (2018). The demographic history of Atlantic salmon (Salmo salar) across its distribution range reconstructed from approximate Bayesian computations*. *Evolution*, *72*(6), 1261-1277. https://doi.org/10.1111/evo.13486

Rougeux, C., Bernatchez, L., & Gagnaire, P.-A. (2017). Modeling the Multiple Facets of Speciation-with-Gene-Flow toward Inferring the Divergence History of Lake Whitefish Species Pairs (Coregonus clupeaformis). *Genome Biology and Evolution*, *9*(8), 2057-2074. https://doi.org/10.1093/gbe/evx150

Roux, C., Tsagkogeorga, G., Bierne, N., & Galtier, N. (2013). Crossing the Species Barrier: Genomic Hotspots of Introgression between Two Highly Divergent Ciona intestinalis Species. *Molecular Biology and Evolution*, *30*(7), 1574-1587. https://doi.org/10.1093/molbev/mst066

Roux, Camille, Castric, V., Pauwels, M., Wright, S. I., Saumitou-Laprade, P., & Vekemans, X. (2011). Does Speciation between Arabidopsis halleri and Arabidopsis lyrata Coincide with Major Changes in a Molecular Target of Adaptation? *PLoS ONE*, *6*(11), e26872. https://doi.org/10.1371/journal.pone.0026872

Roux, Camille, Fraïsse, C., Romiguier, J., Anciaux, Y., Galtier, N., & Bierne, N. (2016). Shedding Light on the Grey Zone of Speciation along a Continuum of Genomic Divergence. *PLOS Biology*, *14*(12), e2000234. https://doi.org/10.1371/journal.pbio.2000234

Tajima, F. (1989). Statistical Method for Testing the Neutral Mutation Hypothesis by DNA Polymorphism. *Genetics*, *123*(3), 585-595.

Tine, M., Kuhl, H., Gagnaire, P.-A., Louro, B., Desmarais, E., Martins, R. S. T., … Reinhardt, R. (2014). European sea bass genome and its variation provide insights into adaptation to euryhalinity and speciation. *Nature Communications*, *5*. https://doi.org/10.1038/ncomms6770
